# Knowledge Graph for Methane Selective Conversion: Revisiting and Predicting Product Selectivity and Methane Conversion

**DOI:** 10.1002/advs.202514601

**Published:** 2025-10-06

**Authors:** Boyu Xu, Gaoyang Li, Bohan Wang, Jiawei Bian, Hui Pan, Yulin Min, Guodong Qi, Jun Xu, Feng Deng, Feng Ju, Hao Ling, Zhendong Wang

**Affiliations:** ^1^ Department of Information and Computing Sciences Utrecht University Princetonplein 5 Utrecht 3584 CC The Netherlands; ^2^ Shanghai Key Laboratory of Materials Protection and Advanced Materials in Electric Power Shanghai University of Electric Power Shanghai 200090 China; ^3^ School of Materials Science and Engineering and East China University of Science and Technology – Utrecht University Joint Research Center for Sustainable and Circular Chemistry and Chemical Engineering East China University of Science and Technology Shanghai 200237 China; ^4^ School of Chemical Engineering and East China University of Science and Technology – Utrecht University Joint Research Center for Sustainable and Circular Chemistry and Chemical Engineering East China University of Science and Technology Shanghai 200237 China; ^5^ National Center for Magnetic Resonance in Wuhan State Key Laboratory of Magnetic Resonance Spectroscopy and Imaging Innovation Academy for Precision Measurement Science and Technology Chinese Academy of Sciences Wuhan 430071 China; ^6^ State Key Laboratory of Green Chemical Engineering and Industrial Catalysis Sinopec Shanghai Research Institute of Petrochemical Technology Co., Ltd. Shanghai 201208 China

**Keywords:** deep neural networks, knowledge graph, large language models, methane selective conversion, predicting catalytic performance

## Abstract

Selective conversion of methane to carbon‐based compounds is promising but currently limited by issues related to scale. Unraveling the interconnected relationships between products selectivity and methane conversion plays a pivotal role in understanding of this complex process. In this work, a knowledge graph (KG) is constructed for methane conversion with the help of a robust large language model basing on the literature reported methane conversion over different catalysts under various conditions. This KG, structured around 11 entity types and 32 relationship types, allows to effectively analyze advancements in methane conversion – including identifying optimal catalytic processes, evaluating reaction conditions, and tracking development trends. A deep neural network analysis of the KG highlighted catalysts with metal active sites and multifunctional supports as particularly effective for methanol production under conditions suitable for industrial‐scale applications. These findings provide valuable insights for targeted catalyst development and industrial applications.

## Graphical abstract

With the help of large language models, deep neural network learning, and kinetic calculation, a knowledge graph prediction of prior research in methane conversion is conducted. This knowledge graph provides a comprehensive understanding of the relationships among catalyst materials, catalytic performance, and reaction conditions, providing guidance for future experimental tasks to accelerate industrial catalyst development.

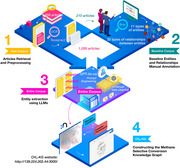

## 1 Introduction

Selective conversion of methane (CH_4_) into carbon‐based compounds is one of the most promising reactions in both nature^[^ ^1^, ^2^, ^3^ ^]^ and the chemical industry.^[^ ^4^, ^5^, ^6^, ^7^ ^]^ Currently, for the concept of low carbon and carbon neutrality, highly efficient methane selective conversion has been widely attended. In catalysis, it is typically performed through water and oxidant reforming, often in conjunction with metal catalysts such as Pd supported metal oxides, Au‐Pd alloy nanoparticles and Au modified zeolites.^[^ ^8^, ^9^, ^10^, ^11^ ^]^ While extensive experimental efforts have been made to develop and validate effective catalysts for methane selective conversion, these trial‐and‐error processes require significant labor from researchers and often face reproducibility challenges. Density Functional Theory (DFT) calculations and simulations have been employed to investigate the detailed properties of catalysts at the molecular level, providing useful insights into catalyst design principles and screening methodologies.^[^ ^12^, ^13^, ^14^, ^15^ ^]^ Beyond using DFT to elucidate reaction mechanisms and interpret experimental phenomena, Artificial Intelligence (AI) has integrated Large Language Models (LLMs)^[^ ^16^ ^]^ with vector‐based statistical modeling,^[^ ^17^ ^]^ Machine Learning (ML), and Deep Learning (DL) techniques to extract and analyze experimental information embedded in a large number of catalytic publications.^[^ ^18^, ^19^, ^20^ ^]^ This integration has facilitated the creation of knowledge networks capable of predicting chemical reactions. The Knowledge Graph (KG) provides an intuitive approach to visualizing information that elucidates the complex relationships, contextual sources,^[^ ^21^, ^22^ ^]^ and transforms textual information into a structured knowledge network.^[^ ^23^, ^24^ ^]^ The key step in constructing the knowledge graph involves text mining and information retrieval tasks.^[^ ^25^ ^]^ The combination of Natural Language Processing (NLP) and LLMs has shown potential in this step, such as text mining for metal‐organic framework synthesis conditions^[^ ^26^ ^]^ and compiling corpora of CO_2_ reduction electro‐catalysts and synthesis procedures.^[^ ^27^ ^]^

The strategy of utilizing Deep Neural Networks (DNNs)^[^ ^28^ ^]^ – a class of machine learning algorithms designed to mimic the brain's information processing capabilities^[^ ^29^ ^]^ – within the established knowledge graph effectively uncovers complex relationships within data.^[^ ^30^ ^]^ DNNs support the extraction and learning of key features from data, enabling pattern recognition and prediction.^[^ ^31^, ^32^ ^]^ For example, DNNs have been used to learn and predict chemical synthesis pathways^[^ ^33^ ^]^ and to identify higher‐selectivity catalysts for known coupling reactions, such as imines and thiols catalyzed by chiral phosphoric acid compounds.^[^ ^34^ ^]^ Deep learning has also been employed to predict Faradaic efficiency in the copper‐based electrocatalytic CO_2_ reduction process.^[^ ^35^ ^]^ Constructing a knowledge network through the aforementioned AI technologies offers an effective approach for a bird's‐eye view of a large number of publications, thereby facilitating comprehensive understanding of the experimental process involved in methane selective conversion. For example, reasonable conditions for non‐oxidative conversion of methane to C_2_ products with low coke selectivity have been predicted by Kim et al. using AI methods.^[^ ^36^ ^]^ Experimental verification has shown that the suggested conditions can indeed produce a considerable C_2_ yield with low coke formation, demonstrating the great potential of AI technologies in predicting methane conversion. This method enables the extraction of useful experimental information while uncovering potential influencing factors and patterns in product selectivity and methane conversion. Although AI technologies, including NLP, LLMs, and DNNs, have been applied in some chemical synthesizes and catalytic reactions, their application in establishing a knowledge network for methane selective conversion remains limited and challenging. This is because methane selective conversion includes the surface adsorption of the oxidant, the activation C‐H bond of methane and the controlling of over‐oxidation, respecting a more complex reaction network than other reactions like CO_2_ reduction. The existing models are inadequate for analyzing the complexities of methane selective conversion and fall short of effectively predicting such dynamic and interfacial processes.

In this work, we extract 11 entity types and 32 corresponding relationship types from 1059 methane selective conversion articles (1987‐2024) using a large language model, GPT‐4o‐ca. Based on this, we develop the methane conversion knowledge graph (CH_4_‐KG) system to integrate experimental data and train predictive models. By leveraging semantic matching to identify highly relevant data, the system predicts product selectivity and conversion rates, while offering an interactive interface to explore patterns and validate predictions efficiently. Moreover, a DNN‐KG model is employed to predict the catalytic performance of various catalysts. In combination with the DNN‐KG method and kinetic calculation, the relationship between the catalytic performance and reaction conditions is conducted to predict the catalytic performance under the reaction conditions, which provides crucial insights to guide future experimental tasks.

## 2 Results and Discussion

### 2.1 Constructing the Knowledge Graph for Revisiting the Methane Selective Conversion

**Figure 1:**
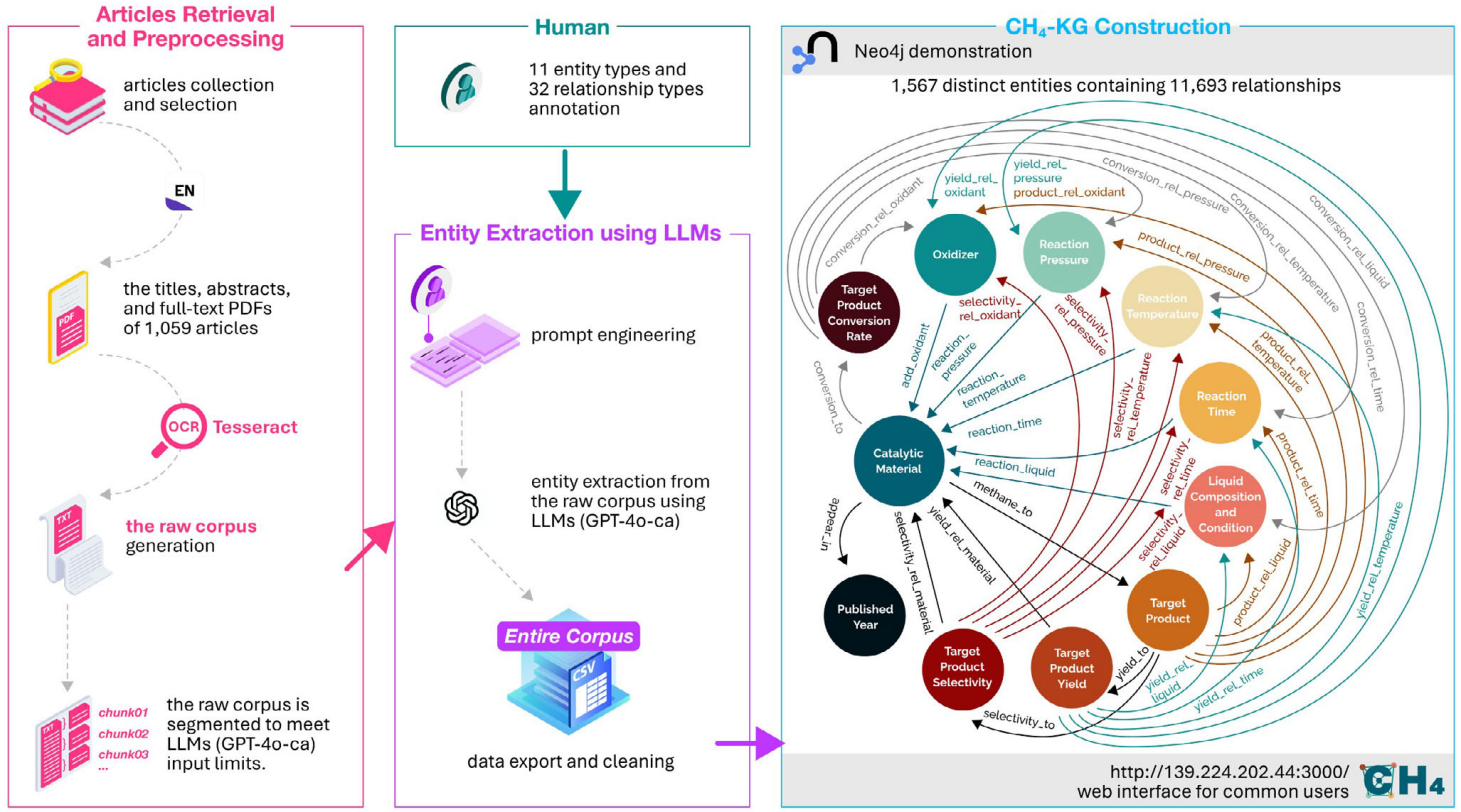
Strategy for constructing the methane selective conversion knowledge graph. Outlines the collection of the raw corpus and the manual annotation of entities and their relationships in methane selective conversion. Explains the generation of the entire corpus by retrieving entities using GPT‐4o‐ca. Presents the construction of CH_4_‐KG in Neo4j and the development of a user‐friendly interface.

The construction of the knowledge graph^[^ ^37^ ^]^ to revisit methane selective conversion, predict product selectivity and methane conversion consists of four components: article retrieval, preprocessing, human manual annotation of baseline entities and relationships, entity extraction using LLMs, and knowledge graph construction (Figure 1 and more details in Experimental Section). For article retrieval and preprocessing, we identified a final collection of 1059 articles focused on thermal catalytic methane selective conversion. We then manually annotated 11 types of entities^[^ ^38^ ^]^ and 32 types of relationships^[^ ^39^ ^]^ to clarify the relationship between reaction conditions and catalytic performance in the methane selective conversion (see Section 4.1). In addition to “Catalytic Material”, “Target Product” and theirs “Published Year”, the “Oxidizer”, “Reaction Pressure”, “Reaction Temperature”, “Reaction Time”, and “Liquid Composition and Condition” were identified as reaction conditions, while “Target Product Selectivity”, “Target Product Yield”, and “Target Product Conversion Rate” were categorized as catalytic performance. We finally identified a total of 1567 distinct entities containing 11693 relationships using LLMs, with rigorous data filtering and cleansing processes applied to 1059 scholarly articles related to methane conversion, thereby constructing the entire corpus (see Section 4.2). To construct the knowledge graph for methane conversion (CH_4_‐KG), the extracted entities and corresponding relations are imported into the Neo4j^[^ ^40^, ^41^ ^]^ graph database platform. The CH_4_‐KG can provide us a sharper overview of methane conversion over various catalysts. This allows us to explore the relationships among “Catalytic Material”, “Target Product”, “reaction conditions”, and “catalytic performance” using Cypher^[^ ^42^ ^]^ queries. We then developed a user‐friendly, code‐free web interface for the CH_4_‐KG (http://139.224.202.44:3000/), its functionalities and visualizations are described in Note S1 and Figure S1 (Supporting Information). This interface allows the researchers, who are not familiar with AI technologies, to use the CH_4_‐KG easily. For example, a drop‐down menu enables a more granular focus by applying multiple limiting conditions simultaneously. The CH_4_‐KG significantly reduces the time and resources required in understanding of methane selective conversion and provides new insights for the design of highly active catalysts and development of new catalytic processes.

### 2.2 Tracking Development Trends in Methane Selective Conversion

**Figure 2:**
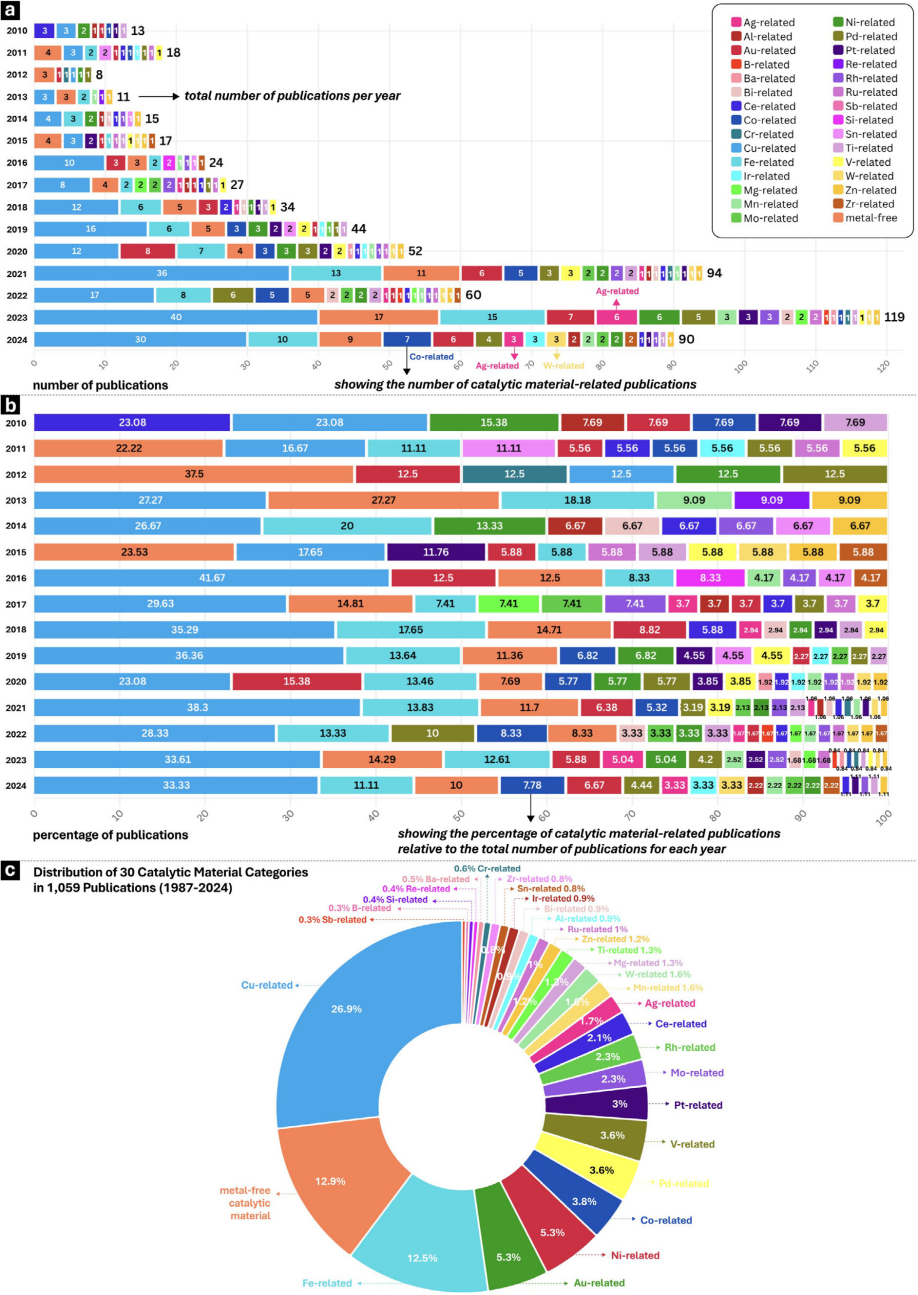
Tracking development trends in methane selective conversion. a) Exploring the yearly distribution of the number of catalytic material‐related publications. b) Exploring the percentage of catalytic material‐related publications within the total annual publications. c) Distribution of 30 catalytic material categories in 1059 publications (1987‐2024).

By extracting entities from the CH_4_‐KG, such as publication year, catalytic materials, and target products, we can systematically revisit and track the development in this field over the past 30 years, rather than focusing on individual research. The yearly publications in the methane selective conversion and their proportions regarding catalytic materials are illustrated in **Figure** **2**a,b. In the first decade (1987‐1997), fewer than ten articles were published annually. In recent years (2020‐2024), the number of publications related to methane selective conversion has grown rapidly, reaching ≈100 articles per year. The investigated catalytic materials include 30 categories basing on the active constituents, such as Cu‐related, Fe‐related, Pd‐related, Ni‐related, and metal‐free catalytic materials. The percentage of publications classified by catalytic materials is summarized in Figure 2c. The results indicate that low‐priced metal (such as Cu, Fe, Ni, and V) containing catalysts and metal‐free catalytic materials (such as boron‐ and carbon‐based materials) are commonly used in methane selective conversion, dominating around 61% of the total publications. Cu‐related catalysts hold significant promise among these catalysts, appearing in ≈27% of all publications. The dynamic progress in methane conversion is visually presented in Figure S2 (Supporting Information) through an animation showing the development of 30 categories of catalytic materials since 1987. This visualization is helpful for identifying newly introduced catalytic materials as well as tracking previously developed ones that have recently been mentioned again. Co‐related and W‐related catalytic materials were first discussed in 1998 and have continued to attract attention over the past five years (2020‐2024). Taking the publications in 2024 for example, seven articles mentioned Co‐related catalytic materials and three articles mentioned W‐related catalytic materials. Ag‐related catalytic materials first emerged in 2017 and have gained increasing attention with nine publications in the past two years (2023 and 2024). The Co‐, W‐, and Ag‐based catalysts have lower prices, higher abundance, and better environmental profile comparing with other metal‐based catalysts. These trigger the re‐attention of such above catalysts, indicating that methane conversion is moving toward a more sustainable and environmentally friendly direction, especially in terms of catalyst design.

**Figure 3:**
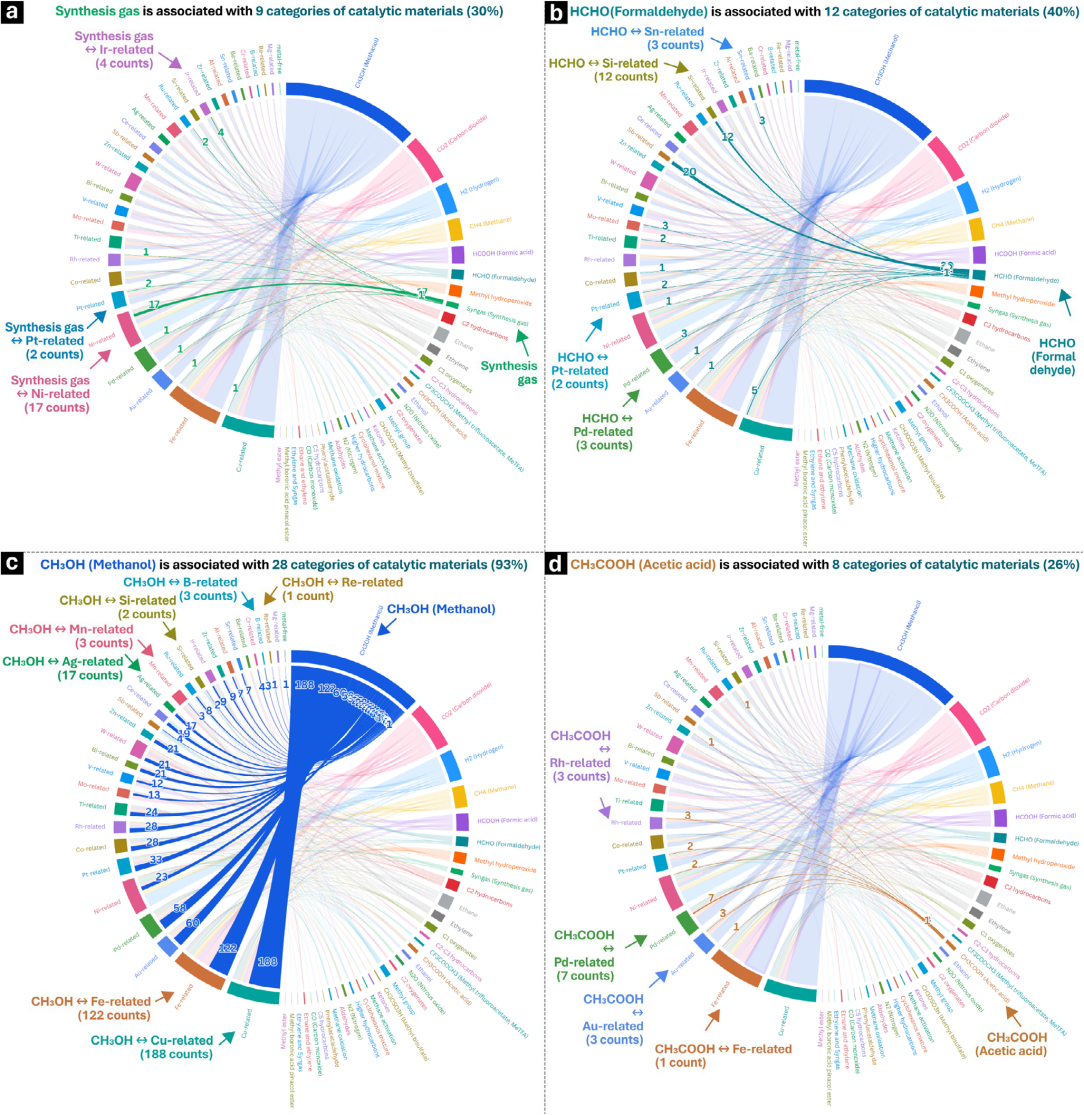
The link between main target products and catalytic materials in methane selective conversion. a) Synthesis gas is associated with 9 categories of catalytic materials, such as Ir‐related and Pt‐related materials. b) Formaldehyde is associated with 12 categories of catalytic materials, such as Si‐related and Sn‐related materials. c) Methanol is associated with 28 categories of catalytic materials, such as B‐related and Mn‐related materials. d) Acetic acid is associated with 8 categories of catalytic materials, such as Rh‐related and Pd‐related materials. The chord diagram is available at: https://public.flourish.studio/visualisation/25051337/.

To explore the influence of catalytic materials on target products, we visualize the relationships between 30 catalytic materials and 34 target products by showing their relationship frequencies. As showed in **Figure** **3**, synthesis gas, formaldehyde, methanol, and acetic acid are the four main target products most strongly linked to catalytic materials in methane selective conversion, corresponding to 9, 11, 28 and 8 categories of catalytic materials, respectively. This provides users with insights for product‐oriented catalytic materials screening or design and vice versa. Methane partial oxidation^[^ ^43^ ^]^ to synthesis gas is energetically a favorable process, which is valuable for downstream processes like Fischer‐Tropsch synthesis, methanol production, or ammonia synthesis. However, the partial oxidation approach requires highly active Ni‐, Pt, or Ir‐ related catalysts under careful control of the oxygen‐to‐methane ratio to ensure partial oxidation rather than complete combustion or explosions. Moreover, this approach often results in a great deal of CO_2_ production as the byproduct, not conforming to the low‐carbon initiatives in modern society (see Figure 3a; Figure S3, Supporting Information). Formaldehyde and methanol are the early hydrocarbon products of methane direct oxidation. To produce formaldehyde, several catalytic materials, including Pd, Sn, and Pt, should be considered because they exhibit similar potential (see Figure 3b; Figure S4, Supporting Information). The first catalytic material for direct oxidation to methanol was an Ag‐based catalyst, discovered in 1987. Since then, various catalytic materials have been developed to catalyze methane oxidation to methanol (see Figure 3c; Figure S5, Supporting Information). Among them, the Si‐related materials are included, which have been first reported for the production of formaldehyde in 1995, and subsequently, this kind of material has been found having activity in conversion of methane to methanol in 2016. In recent years, particularly in 2021 and 2023, many reports have shown that Cu‐ and Fe‐related materials exhibit high activity in the partial oxidation of methane to produce methanol using O_2_ as an oxidant. Moreover, when searching for or designing catalytic materials that produce CH_3_OH, less‐explored materials such as Re‐, B‐, and Mn‐related materials could provide additional options alongside the well‐known Cu‐ and Fe‐related materials. These less‐explored catalytic materials have not been extensively studied in experiments so far, and perhaps they require further attention. Acetic acid is one of the most valuable products for methane conversion, it was first produced in methane conversion over RhCl_3_ catalyst in 1994. Nowadays, the catalytic oxidation of methane to value‐added C2 products (e.g., acetic acid) has attracted wide attention, focusing on Rh‐ or Pd‐based homogeneous catalysts and Fe‐ or Au‐related zeolitic heterogeneous catalysts (see Figure 3d; Figure S6, Supporting Information). Some catalytic materials can produce multiple target products within the same reaction system, thereby improving experimental efficiency. For example, 12 categories of catalytic materials, including Cu‐related and Sb‐related materials, are associated with the production of both methanol and formaldehyde (see Figure S7, Supporting Information), while 7 categories of catalytic materials, such as Ni‐related and Pd‐related materials, are associated with the production of both synthesis gas and acetic acid (see Figure S8, Supporting Information). Methane selective conversion is a complex process that requires careful catalyst design and optimization. Homogeneous catalysts offer high selectivity but face challenges in catalyst recovery, while heterogeneous catalysts provide easier separation but often suffer from lower selectivity. Additionally, the Fe‐related catalytic materials also demonstrate the capability to generate C3 and C5 oxygenates. This indicates that the Fe molecular structure plays a role, making it worth further exploration.

### 2.3 Analyzing Reaction Conditions in Methane Selective Conversion

**Figure 4:**
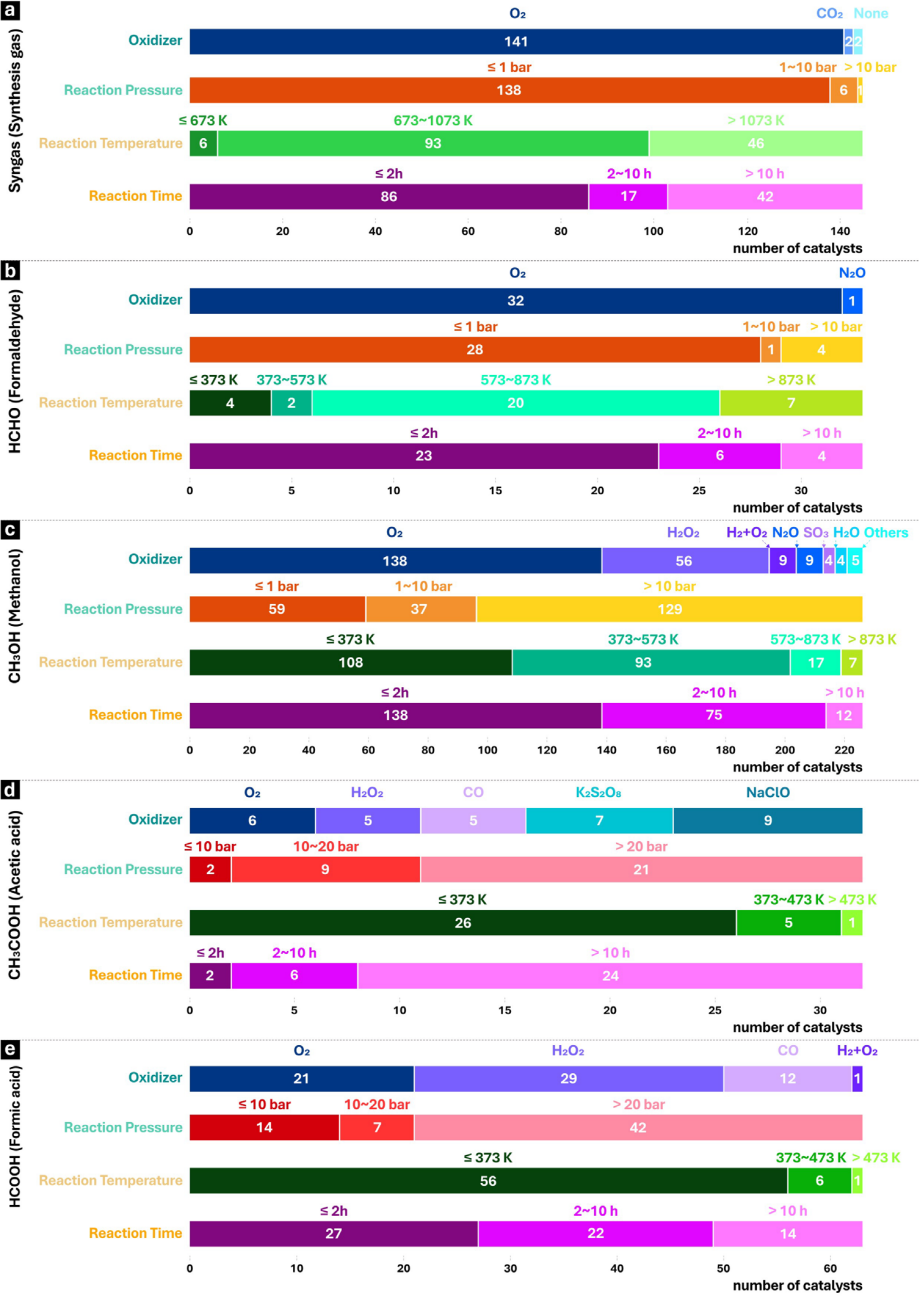
The reaction conditions (detailing oxidizer, reaction pressure, reaction temperature, and reaction time) for the selective conversion of methane to the target products: a) synthesis gas, b) formaldehyde, c) methanol, d) acetic acid, and e) formic acid.

Reaction conditions, including oxidants, temperature, pressure, and gas‐/liquid‐phase affect the product distribution and selectivity significantly. Based on literature norms or application benchmarks, mild conditions are defined as a temperature below 500 K and pressure below 10 bar, moderate conditions as a temperature of 500–700 K and pressure of 10–20 bar, and harsh conditions as a temperature above 700 K and pressure above 20 bar. The reaction conditions for selective conversion of methane to produce formaldehyde, syngas, organic acids and methanol are illustrated in Figure 4 and Figures S9–S13, Supporting Information. As shown in Figure 4a and Figure S9 (Supporting Information), more than one hundred reports have shown that syngas synthesis via CH_4_ and O_2_ predominantly occurs in high‐temperature (above 1000 K) conditions under atmospheric pressure with high efficiency. High reaction pressure is unnecessary for syngas synthesis, more than 95% of the catalysts show reactivity under ambient pressure. The complete combustion or explosions are key issues for this reaction if the CH_4_ and O_2_ ratio is not carefully controlled. Oxygen also serves as the primary oxidizer for formaldehyde production over more than twenty catalysts (see Figure 4b; Figure S10, Supporting Information). The reaction can be operated both under high‐ and low‐pressure conditions, however, high reaction temperature (generally below 873 K) is usually required. Given these conditions, short reaction (contact^[^ ^44^ ^]^) time is beneficial for increasing product selectivity by inhibiting over‐oxidations.^[^ ^45^ ^]^ More than 70% of the catalysts are operated for reactions within two hours for formaldehyde production. Selective conversion of methane to methanol is the dream reaction in catalysis.^[^ ^46^ ^]^ Great efforts have been made and almost all of the available oxidants have been employed to achieve this reaction (see Figure 4c; Figure S11, Supporting Information). From an economic point of view, oxygen is the cheapest oxidant and easy to get, while H_2_O_2_ perhaps is the most expensive one. These two oxidants are environmentally friendly because they will be transformed into water after the reaction. Therefore, O_2_ and H_2_O_2_ have been considered as the first and second preferred oxidant, respectively. Many reaction models including solid‐gas, liquid–gas and solid–liquid–gas reactions, can be chosen in oxidation of methane using O_2_. The reaction conditions vary significantly depending on the reaction model and the nature of the catalyst. Oxidation of methane to methanol using H_2_O_2_ is often operated in aqueous medium (water); high reaction pressure (higher than 10 bar) is necessary due to the low solubility of methane in water, sometimes with additives to enhance methane solubility. Almost half of the catalysts are optimized under mild to moderate reaction temperatures (below 573 K) to balance reaction rate and enhance methanol selectivity. Short reaction (contact) times or continuous extraction of methanol to prevent excessive over‐oxidation are suggested for the reaction using both O_2_ and H_2_O_2_. When targeting organic acids as end products, the choice of appropriate oxidants and reaction pressure becomes essential (see Figure 4d,e; Figures S12 and S13, Supporting Information). Acetic acid formation typically utilizes various oxidants, such as NaClO, O_2_ and K_2_S_2_O_8_, H_2_O_2_, and CO, while formic acid synthesis is only operated using O_2_ or H_2_O_2_ as the oxidant. Although moderate temperatures (typically around 353 K) are sufficient for the conversion of methane to these acids, high reaction pressure and long reaction time are required to achieve the C‐C coupling and carboxylation steps in the reaction, which eventually cause low selectivity due to over‐oxidation.

### 2.4 Predicting Product Selectivity and Methane Conversion

**Figure 5:**
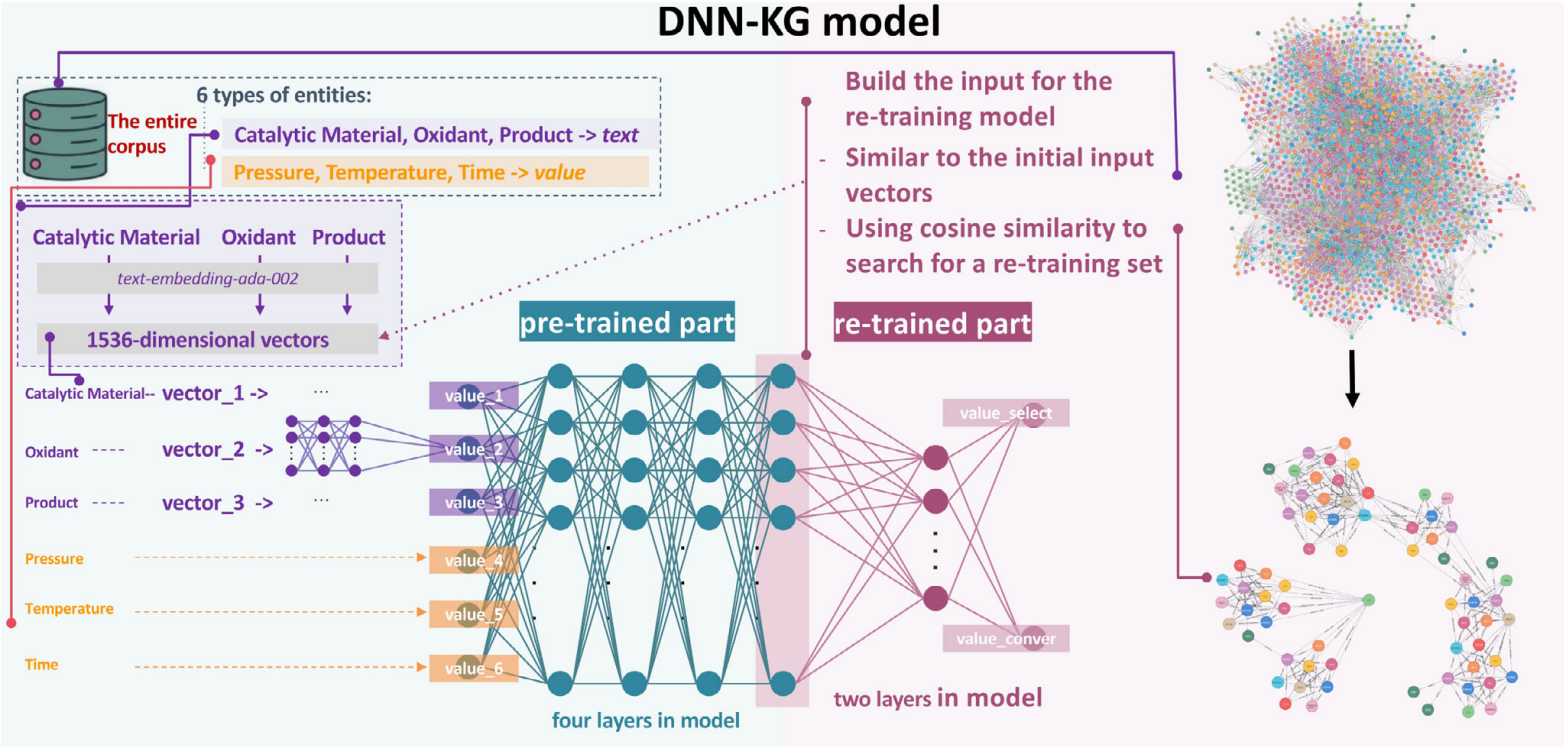
Overview of the DNN‐KG Model. The model's input consists of six entity types: three text‐based entities (Catalytic Material, Oxidizer, and Target Product) and three numerical entities (Reaction Pressure, Reaction Temperature, and Reaction Time). The text‐based entities are transformed into 1536‐dimensional vectors using the text‐embedding‐ada‐002 model, while the numerical entities are used directly as values. These together form the input features for the DNN‐KG model. The model is composed of a four‐layer pre‐trained section and a two‐layer re‐trained/fine‐tuning section. By leveraging the knowledge graph, the model uses cosine similarity to search for data similar to the initial input vectors, thus constructing a re‐training dataset and forming localized knowledge graphs.

The CH_4_‐KG reveals that optimal reaction conditions for methane conversion to target products are highly variable, as they are highly sensitive to the oxidant, catalyst type, and solvent properties. As a fundamental application of deep learning, the DNN is constructed by stacking multiple fully‐connected layers. Through this basic framework, the DNN possesses high plasticity and potential, and many network architectures, including Physics‐Informed Neural Networks (PINNs), are derived from it.^[^ ^47^ ^]^ The “deep” multi‐layer structure enables the DNN model to learn hierarchical features from the concrete to the abstract and endow it with end‐to‐end learning capabilities. It means that the DNN model can automatically induce effective features for learning simply by being provided with raw data.^[^ ^29^ ^]^ Therefore, the DNN is capable of integrating heterogeneous input data and extracting feature information from high‐dimensional word vectors, making it an ideal and powerful tool for unravelling the complex relationships between catalytic performance, catalytic materials and reaction conditions in our current study (see Section 4.4). Thereupon, the CH4‐KG data were used to train and build a CH_4_‐KG and DNN coupling model (DNN‐KG) to predicting product selectivity and methane conversion under various conditions and catalytical materials. As revealed in **Figure** **5**, we integrate textual information into DNN model through leveraging the identified entities and text embedding techniques. The cosine similarity between the input and the nodes in the corpus is calculated to identify nodes with the highest similarity. The relational data within the CH_4_‐KG enables us to map catalysts and conditions to their corresponding products, thereby creating the training set for the DNN. The re‐training architecture gives a provision for integrating private databases. Moreover, the unique pre‐training and re‐training data provide the correlation of similar entries in the CH_4_‐KG, allowing the DNN analysis to focus on specific catalyst types to enhance its predictive accuracy (seeing the comparison with traditional approach in Figure S14, Supporting Information for details).

The above‐established DNN and CH_4_‐KG coupling model (DNN‐KG) is employed to predict the methane oxidation to methanol performance over different catalytic materials under various reaction conditions. We analyzed the predicted selectivities and conversions of methane oxidation to methanol in the temperature range from 300 to 800 K under 1 to 30 bar, using various catalytic materials including zeolite‐, transition metal‐, noble metal‐, and metal salt‐based catalysts. Table S1 (Supporting Information) presents a comparison of predicted and reported methane conversion and methanol selectivity for five randomly selected catalysts, showing the consistency between the reported values and the model predictions. To overcome the difficulty in comparison of various catalysts, a simple kinetic model was employed to describe the selectivity‐conversion trade‐off for these catalysts.^[^ ^48^ ^]^

(1) $$S=\frac{1-X-{\left(1-X\right)}^{k_{2}/k_{1}}}{X*\left(k_{2}/k_{1}\right)}$$

where S is the selectivity, X is the conversion, k_1_ and k_2_ are the microscopic rate constants in reaction Equation (2), which are calculated by the difference in methane and methanol activation free energies, ΔG and the temperature T in Equation (3), where k_b_ is the Boltzmann constant and ΔG is approximated in the remainder of this work as its linear best fit with the electronic activation energies *Δ*E in Equation (4).

(2) $$CH_{4}\overset{k_{1}}{\to }CH_{3}\mathrm{OH}\overset{k_{2}}{\to }CO_{2}$$

(3) $$k_{2}/k_{1}=e^{\Delta G/k_{b}T}$$

(4) $$\Delta G\;=\Delta E-\left(3.942\times 10^{-4}\right)\cdot T-0.0289$$

**Figure 6:**
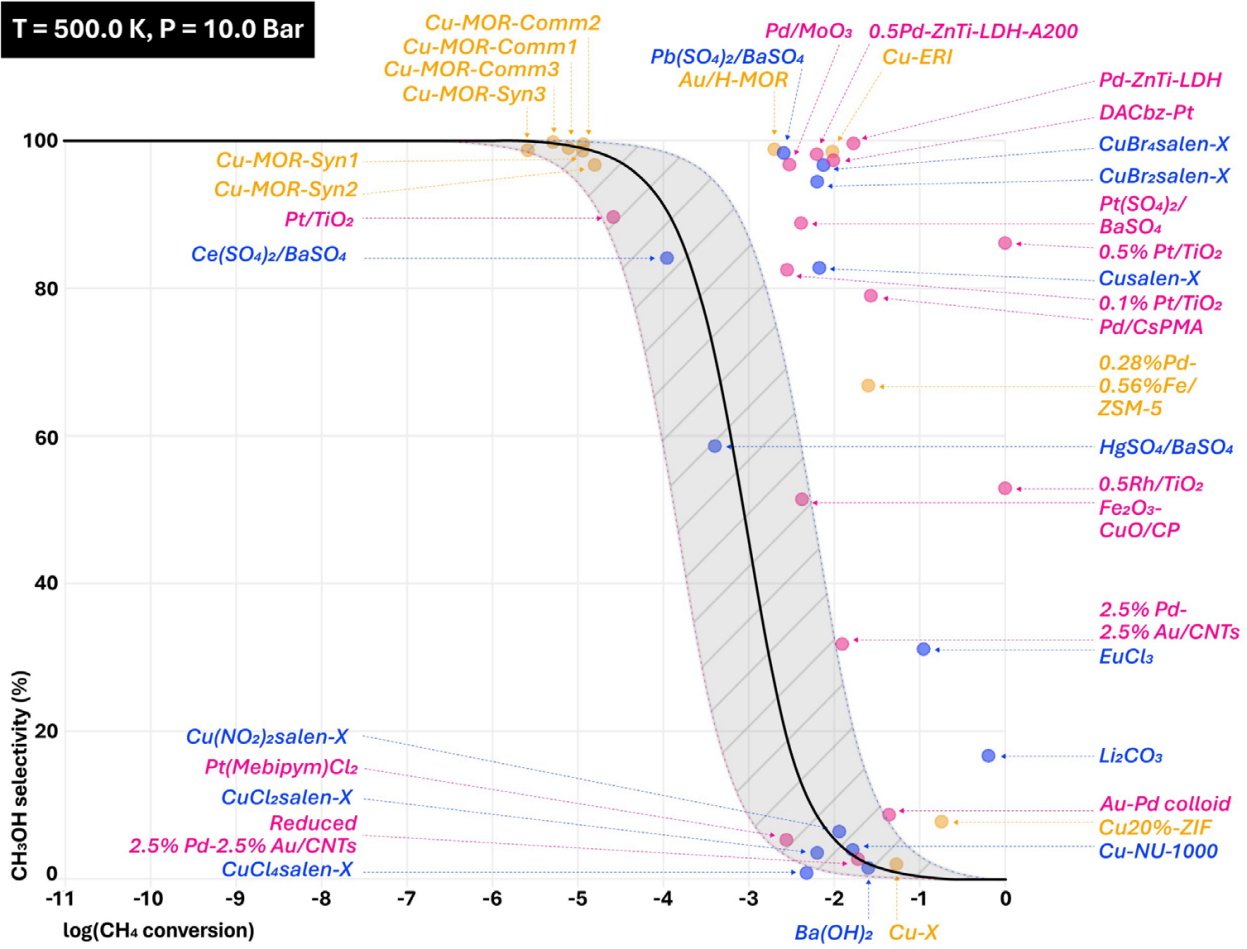
Predicted CH_3_OH selectivity and CH_4_ conversion at 500.0 K under a total reaction pressure of 10.0 bar. Zeolites‐ and MOFs‐based catalysts (orange dots), transition metal‐ and metal salt‐based catalysts (blue dots) and noble metal‐based catalysts (pink dots) overlaid on the non‐catalytic selectivity‐conversion trade‐off line described by Equation (1) at 500 K under 10.0 bar using ΔG_DFT_ (black line) including a ±1 σ error (the shading area between blue and pink dash lines).

As showed in Figures S15–S20 (Supporting Information), a natural exponential function was used to plot these predicted data (including catalysts, conditions, and product outcomes), along with the calculated non‐catalytic selectivity‐conversion trade‐off line from ΔE and Equation (1) under specific conditions with a 0.55 ± 0.08 range (see Section 4.4 for details). As shown in Figure S15 (Supporting Information), almost all the predicted data are in the region away from the non‐catalytic line at 300 K under 1 to 30 bar owing to the catalytic role of these materials. Some zeolite‐, noble metal‐ and metal salt‐based catalysts, such as Cu/ERI, Pd‐ZnTi‐LDH and CuBr‐salen, appear in the right‐top corner of the graph, demonstrating their excellent catalytic performance under this condition. The increase of reaction pressure from 1 to 30 bar at 300 K leads to a gathering of data points in the right‐top corner of the graph. This finding implies the promoting effect of reaction pressure on the catalytic activities of these catalysts. It is worth noting that the reaction pressure significantly influences the performance of zeolite‐ and noble metal‐based catalysts, like Pd‐Fe/ZSM5 and Pt/TiO_2_. Increasing the reaction temperature under 1 bar causes the data for some transition metal‐ and metal salt‐based catalysts, for example, Cu‐X and EuCl_3_, gradually fall into/down the non‐catalytic region. This phenomenon grows significantly more pronounced under synchronous increases in reaction temperature and pressure. Some zeolite‐ and noble metal‐based catalysts, for instance Cu‐MORs and Pt/TiO_2_, are found to lose their catalytic role although they exhibit excellent catalytic performance at mild conditions. According to the literature studies, harsh conditions can change the structure of the catalyst (e.g., pore collapse, metal agglomeration, or surface oxidation), leading to deactivation. This may also arise from the complex interplay of reaction kinetics, catalyst properties, and process conditions. Harsher conditions drive the reaction toward over‐oxidation or undesired side reactions, increasing conversion but reducing selectivity. From the perspective of long‐term operation or industrial conditions, catalysts under mild operating conditions and high activity have the greatest application potential. Figure 6 illustrates the predicted catalytic performance of the catalysts in methane conversion under mild pressure (10 bar) and moderate temperature (500 K), with the consideration of the practicality of using such conditions in industrial processes. These catalysts are distributed into different regions based on selectivity and methane conversion. In the high selectivity region located close to the upper apex of the figure, there are Au/H‐MOR, Pb(SO_4_)_2_/BaSO_4_, Pd/MoO_3_, Pd‐ZnTi‐LDHs, Cu‐ERI, and Cu‐MORs catalysts, with nearly 100% product selectivity under such mild conditions. However, despite their high selectivity, these catalysts exhibit low methane conversion, less than 1%. This suggests that while they are effective in ensuring the desired products, their efficiency in converting methane is limited. In contrast, the high methane conversion region at the far right of the figure contains Pt/TiO_2_, Rh/TiO_2_, and Li_2_CO_3_ catalysts. These catalysts achieve almost complete methane conversion with product selectivity of 86.13%, 52.90%, and 16.66%, respectively. The catalysts in the rest region of this figure perform low methane conversion and unremarkable product selectivity, unsatisfying the necessary thresholds for industrial conditions. This indicates a balance between high conversion rates and maintaining selectivity, which is crucial for industrial applications where both efficiency and product quality are paramount. These findings highlight the selectivity and conversion trade‐off in catalysis, which is vital for developing catalysts that meet the stringent requirements for long‐term operation and industrial processes. It can also be supposed that noble metal Au‐, Pt‐, Pd‐, or transition metal Cu‐based active phase combined with a multi‐functional support, like a complex of metal oxide (TiO_2_, MoO_3_, etc.) and zeolite (MOR, ERI, etc.), may have a good potential for high selectivity and methane conversion. However, the suitable reaction conditions need to be further studied. In summary, our predictions demonstrate a comparative analysis of catalyst performance, offering insights into the complexities of methane conversion and the potential for future development and application in this field. Furthermore, some of the conditions in our prediction are challenging and promising to achieve experimentally, like the extremely high temperature and pressure, thereby reducing research costs in terms of time and human resources.

## 3 Conclusion

We construct a methane knowledge graph (CH_4_‐KG) by analyzing 1059 research articles in methane selective conversion published over the past two decades. Utilizing the capabilities of Large Language Models (LLMs) in natural language understanding and large‐scale information processing, we systematically extract 11 entity types and 32 corresponding relationship types relevant to methane selective conversion. This CH_4_‐KG provides a structured visualization of complex relationships among catalytic materials, reaction conditions, target products, catalytic performance and etc. It not only significantly reduces the time and effort required for comprehensive literature reviews, but also offers a clearer overview of reaction processes, supporting researchers in identifying trends and promising catalytic systems. To enhance usability, we integrate the KG into an interactive, user‐friendly interface. This interface allows users to identify the correlations between catalytic materials and reaction conditions. Ours analysis shows that the optimal reaction conditions for methane conversion to target products remain uncertain, as they are highly dependent on the oxidant, the type of catalyst, and solvent properties. Driven by internationally recognized carbon neutrality, renewable energy and climate adaptation for social development, methane conversion is shifting from a “high‐carbon” process to a “low‐carbon and value‐added” process and becoming increasingly important. We then train a Deep Neural Network (DNN) and CH_4_‐KG coupling model (DNN‐KG) in combination with kinetic calculation to predict the catalytic performance of methane oxidation to methanol over various catalysts under different reaction conditions. The DNN‐KG provides the structured linked data, enabling the mapping of catalysts and conditions to corresponding products, which indicate that catalysts featuring of metal active sites and multifunctional supports characteristics possibly exhibit high activity under mild conditions suitable for industrialization.

This study bridges structured knowledge representation with predictive modelling, offering hypotheses to guide experimental design in methane selective conversion, however, requiring of experimental validation. Our approach provides a concrete case study for extending LLMs‐augmented KG and DNN frameworks to broader catalytic research in methane conversion, it has the potential, not yet been applied to other reactions. The incorporation of LLMs facilitates scalable, automated knowledge extraction in the catalytic research. The coupled DNN‐KG model helps mitigate the reliance of data‐driven models on large, high‐quality datasets and enhances their transferability across catalytic contexts. LLMs often have model hallucination problem in specialized domains, we train the DNN‐KG model to avoid this issue. Moreover, fine‐tuning LLMs with expert knowledge Question and Answer (Q&A) databases and retrieval‐augmented methods can also mitigate hallucination, providing more professional guidance. While the use of a DNN‐KG model is well‐motivated, it is noted that the application of AI in methane conversion is still limited. Looking to the future, a more powerful professional agent for catalytic research can be expected, which leverages LLMs as its core to process complex catalytic knowledge, enabling the integration with simulation software, like Model Context Protocol (MCP) and Density Functional Theory (DFT). Such specialized agent will be able to achieve the automated data retrieving and updating from both experiments and online literatures, thereafter, aiding intelligent optimized design of catalytic experimental research.

In summary, our strategies, including CH_4_‐KG construction, DNN predication and kinetic calculation, have the potential to enhance our knowledge in methane selective conversion. The results will be helpful in bringing future large‐scale implementation of methane conversion, addressing key bottlenecks such as catalyst life‐span, scale‐up, emissions, and cost efficiency. Expanding the scope of this DNN‐KG approach is a promising avenue for future research. The demonstrated success with methane conversion suggests a robust framework that could be readily adapted to other catalytic reactions, such as electro‐ and photo‐catalytic systems.

## 4 Experimental Section

### Article Retrieval and Entities and Relationships Annotation

The articles on methane selective conversion, published between January 1987 and June 2024, were retrieved through a multi‐stage filtering process. Boolean operators^[^ ^49^ ^]^ were used to search articles related to methane selective conversion in the Web of Science Core Collection, see Note S2 (Supporting Information). The search criteria include “Methane Selective Conversion” (Topic), “Methane to Methanol” or “Methane Selective Oxidation” (Keyword), “Article” (Document Type), and “English” (Language), yielding 3402 articles as of 28 June 2024. Articles were first filtered with titles containing “methan*”, “CH4”, or “CH(4)”. Then it conducted a round of manual screening to exclude articles whose contents were theoretical studies and not directly relevant to the topic, such as those containing terms like “microbial”, “electric”, “solar”, or “light”. This filtering process resulted in a final collection of 1059 articles focused on experimental studies in thermal catalytic methane selective conversion, which provide comprehensive coverage of the subject. Using a semi‐automated approach, the titles, abstracts, and full‐text PDFs were retrieved for these 1059 articles from the web. Tesseract was then employed, an Optical Character Recognition (OCR) tool to extract titles, abstracts, full texts, and figure captions from the PDFs into.txt format, generating the raw corpus.

It defined 11 entity types that were useful for understanding the methane selective conversion experiment. These include “Catalytic Material”, “Oxidizer”, “Reaction Pressure”, “Target Product”, “Published Year”, “Reaction Temperature”, “Reaction Time”, “Liquid Composition and Condition”, “Target Product Yield”, “Target Product Selectivity” and “Target Product Conversion Rate”. For example, “Catalytic Material” refers to the catalytic material(s) used during the methane selective conversion, such as CeO_2_/Cu_2_O/Cu(111). “Target Product” refers to the compounds produced during methane selective conversion, such as methanol. “Target Product Selectivity” refers to the percentage of the desired product relative to the total products formed. It also defined 32 relationship types that map the interactions among these entities, outlining the process of methane selective conversion. A detailed description and examples of each entity type are provided in Table S2 (Supporting Information), and a detailed description of each relationship type is provided in Table S3 (Supporting Information).

To ensure the quality of the training corpus derived from automatically extracted models, a baseline corpus was created, known as a gold standard corpus, by manually annotating entities and corresponding relationships present in the titles, abstracts, and conclusions of 210 articles on methane selective conversion. It used brat,^[^ ^50^ ^]^ a web‐based text annotation tool that facilitates the addition of annotations to existing text (see Note S3, Supporting Information). Annotators first defined the 11 entity types and their corresponding 32 relationship types within the configuration. This manual annotation resulted in 492 entity tags, with the number of tags for each entity type detailed in Table S4 (Supporting Information). These annotations serve as the gold standard corpus for evaluating the automatically extracted models.

### Entity Extraction using Large Language Models

LLMs can assist researchers in improving the efficiency of entity extraction by analyzing extensive text corpora and identifying relevant entities. In this study, it utilize GPT‐4o‐ca^[^ ^51^ ^]^ a model provided by Azure OpenAI. To meet the input constraints of GPT‐4o‐ca, the raw corpus (in.txt format), which comprises titles, abstracts, full texts, and figure captions from 1059 articles, was segmented into manageable text chunks (see Note S4, Supporting Information). This segmentation allows the model to process the corpus effectively while preserving the integrity of the extracted information.

Prompt engineering (PE) was a useful technique for improving LLM performance in entity extraction.^[^ ^52^ ^]^ It enables researchers to customize the LLMs’ responses, directing the model to provide information that aligns with the research goals. For example, customized prompts focused on methane selective conversion enable the model to extract entities such as “Catalytic Material”, “Oxidizer”, and “Target Product”. These prompts can also standardize units, converting temperature to Kelvin (K), pressure to bar, time to hours (h), and selectivity and conversion rates to percentage (%), thus ensuring consistency in the extracted information. An example of entity extraction using GPT‐4o‐ca through prompts is shown in Figure S21 (Supporting Information). The extracted entities were then exported in CSV format using commas as delimiters and undergo a cleaning process to eliminate duplicates and filter out irrelevant or unreadable entries. It also manually clean the data, ensuring that the entities and corresponding relationships were both accurate and contextually consistent. The entity extraction yields a total of 1567 entity tags and 11693 relationship tags, including 335 “Catalytic Material” tags and 40 “Oxidizer” tags. The count and frequency of each entity type in the entire corpus are detailed in Table S5 (Supporting Information).

It evaluate the availability and accuracy of the model's extraction results using 10 of the 210 articles on methane selective conversion (baseline corpus). As listed in Table S6 (Supporting Information), a total of 10 rows of data were automatically extracted, each containing 11 entities, resulting in 96 entries with numerical values and 14 entities with empty values (marked as “none”). The extracted entries were then manually traced to the original publications, which involved comparing the extracted catalytic materials and publication years with those in the baseline corpus, locating the literature DOIs, and reviewing articles to verify the accuracy of the extracted information (see Figure S22, Supporting Information). The evaluation results indicate that the automatic extraction by GPT‐4o‐ca exhibits 87.2% availability and nearly 100% accuracy.

### Constructing the Knowledge Graph

To clarify the relationship between reaction conditions and catalytic performance in the methane selective conversion experiment, it identified “Oxidizer”, “Reaction Pressure”, “Reaction Temperature”, “Reaction Time”, and “Liquid Composition and Condition” as reaction conditions, while “Target Product Selectivity”, “Target Product Yield”, and “Target Product Conversion Rate” were categorized as catalytic performance. The extracted entities and corresponding relationships from 1059 articles were imported into the Neo4j graph database platform to construct the CH_4_‐KG. To provide a user‐friendly, code‐free interface for exploring methane selective conversion, the CH_4_‐KG website (http://139.224.202.44:3000/) was developed, its functionalities are described in Note S1 (Supporting Information).

### Predicting Product Selectivity and Methane Conversion using the DNN‐KG Model

It propose the DNN‐KG method to output values related to product selectivity and methane conversion were predicted to assess the impact of reaction conditions on catalytic performance, thereby guiding future experimental tasks. Plenty of extracted data samples from the 1059 articles were used to develop the constructed knowledge graph and pre‐trained through a DNN model. As depicted in Figure 5, a DNN‐KG model includes pre‐trained and re‐trained sections. To improve prediction accuracy, the cosine similarity between the input and the nodes in the constructed knowledge graph was calculated to identify nodes with the highest similarity to form a small re‐train dataset (less than 4% of the extracted data samples).

Specifically, after manual data cleaning, a total of 603 extracted data samples from the 1059 articles were used to develop the constructed knowledge graph. These samples were then split into a training dataset (80% of the total data samples), a validation dataset (4% of the total data samples), and a test dataset (16% of the total data samples). The training dataset was used to fit the model parameters first, and the re‐trained dataset from the constructed knowledge graph was then used to update the re‐trained model parameters. The validation dataset was used to evaluate the hyperparameter tuning, and the test dataset was used to evaluate the prediction performance. It compared the proposed DNN‐KG model with the traditional DNN model, and Figure S14 (Supporting Information) shows that our method performs well by using a pre‐trained model and retraining it through the small dataset which was formed by capturing dependencies between the input values and adjacent nodes based on the constructed knowledge graph. Moreover, the text embedding in our method establishes information flow between input text and connected nodes in the graph and propagates linguistic representations from vertex to vertex to learn latent representations.

## Conflict of Interest

The authors declare no conflict of interest.

## Author Contributions

B.X. and G.L. contributed equally to this work. B.X., F.J., G.Q., H.P., and G.L. performed conceptualization. G.Q., F.J., and H.P. performed methodology. B.X., G.L., and B.W. performed investigation. B.X., G.L., and J.B. performed visualization. G.Q., F.J., and H.P. performed supervision. B.X., G.L., G.Q., H.P., and F.J. wrote the original draft. B.X., G.L., G.Q., H.P., Y.M., F.J., J.X., F.D., H.L., and Z.W. wrote, review and edited the final manuscript.

## Supporting information



Supporting Information

## Data Availability

The data that support the findings of this study are available from the corresponding author upon reasonable request.
