# Supplementary material for: Knowledge Graph for Methane Selective Conversion: Revisiting and Predicting Product Selectivity and Methane Conversion
Source: Adv Sci (Weinh). 2025 Oct 6;12(48):e14601. doi: 10.1002/advs.202514601 (PMC12752571; doi:10.1002/advs.202514601)
Supplement: Supplementary file 1 — Supporting Information [file ADVS-12-e14601-s001.docx]

Supporting Information

Knowledge Graph for Methane Selective Conversion: Revisiting and Predicting Product Selectivity and Methane Conversion

*Boyu Xu,*† *Gaoyang Li,*† *Bohan Wang,* *Jiawei Bian,* *Hui Pan,** *Yulin Min,* *Guodong Qi,** *Jun Xu,* *Feng Deng,* *Feng Ju,** *Hao Ling,* *and Zhendong Wang*

**1. Supporting Notes (Note S1-S4)**

**Note S1:** Five functionalities provided by CH_4_-KG are outlined below:

- *The Node Count* Display (see **Figure S1**): Users can view the total number of nodes in the graph, as well as the count for each entity type.
- *The Structured Overview of the Selected Entity* (see **Figure S1**): Each entity is visualized as a node. When a user clicks on a node, detailed information about its structure is displayed, showing linked entities and relationships.
- *The Data Filtering* (see **Figure S1**): Users can filter the knowledge graph by selecting one or more specific entities via a dropdown menu.
- *Zoom In/Out* (see **Figure S1**): Users can adjust the knowledge graph’s scale using the mouse scroll wheel to obtain a more detailed or broader view of the data.
- *Save the Current Knowledge Graph* (see **Figure S1**): Users can save the current knowledge graph in JPEG or PNG formats.

Note S2: The scientific publications referenced in this study include journal articles from *Elsevier*, *Wiley*, the *American Chemical Society*, *Springer Nature*, and other prominent publishers.

- We conducted a search for articles on methane selective conversion in the Web of Science Core Collection using the following criteria:
  - “Methane Selective Conversion” (Topic);
  - “Methane to Methanol” or “Methane Selective Oxidation” (Keyword);
  - “Article” (Document Type); and
  - “English” (Language).

This search yielded **3,402 articles as of 28 June 2024**.

- We applied the following selection criteria to screen methane-related articles:
  - Titles containing “methan*”;
  - Titles containing “CH4” or “CH(4)”.

This initial screening reduced the selection to **2,073 articles**.

- We then manually excluded articles with titles not directly relevant to the topic, such as those containing terms like “microbial”, “electric”, “solar”, “light”, “biological”, “enzyme”, or “fuel”, among others.

This filtering process resulted in a final collection of **1,059 articles** focused on thermal catalytic methane selective conversion.

- These 1,059 articles were imported into [EndNote 20](https://endnote.com/?srsltid=AfmBOorh0k-o_5MQKPujX0sIhZXl0433ylrAleWTc1NGoL4eawjybUk) (<https://endnote.com/>) bibliographic database.
- The “Find Full Text” feature in EndNote 20 retrieved the titles, abstracts, and full-text PDFs for 519 articles, while the remaining 540 PDFs were downloaded manually by the authors.
- Tesseract (<https://github.com/tesseract-ocr/tesseract>), an Optical Character Recognition (OCR) tool, was used to extract titles, abstracts, full texts, and figure captions from the PDFs into .txt format, generating the raw corpus.

**Note S3:** Annotators use brat (<https://brat.nlplab.org/>), a web-based text annotation tool, to manually annotate entities and relationships in text.

- Definition: Annotators follow the defined 11 entity types and 32 relationship types in the methane selective conversion experiment. These definitions are configured within brat to guide the annotation process.
- Annotation Process: Annotators open and browse the sentences requiring annotation in a web browser. Each annotator analyzes the sentences and labels the entities and relationships.
- Collaboration and Quality Control: The brat interface allows for collaboration among multiple annotators, ensuring consistency and accuracy in the annotations. To maintain annotation quality, a subset of annotations is cross-checked by multiple annotators, with discrepancies resolved through discussion.
- Exporting Annotations: After manual annotation, annotators can export the annotated entities and corresponding relationships as .ann files. These annotations serve as the gold standard corpus for evaluating the automatically extracted models.

**Note S4:** The entity extraction process using GPT-4o-ca involves the following steps to ensure the efficient and accurate identification of entities from the raw corpus:

- Step 1: Data Segmentation

The raw corpus, which includes titles, abstracts, full texts, and figure captions from 1,059 articles, is segmented into smaller, manageable chunks. This segmentation is necessary to comply with the input constraints of GPT-4o-ca and ensures that the model can process large volumes of text efficiently.

- Step 2: Prompt Engineering

Customized prompts are designed to focus on specific research needs. These prompts are tailored to extract entities such as “Catalytic Material”, “Oxidizer” and “Target Product”, and to standardize units, such as converting temperature to Kelvin (K), pressure to bar, time to hours (h), and selectivity and conversion rates to percentages (%).

- Step 3: Entity Extraction

GPT-4o-ca processes the text chunks and extracts entities based on the predefined prompts.

- Step 4: Data Export and Cleaning

The extracted entities are exported in CSV format with commas as delimiters. We then manually clean the data to ensure accuracy and consistency.

- Step 5: Validation

The extracted entities are compared to a manually annotated baseline corpus for validation. This step involves cross-checking the extracted information, such as catalytic materials and publication years, with the baseline corpus to ensure the accuracy of the extraction.

# 2. Supporting Figures (Figure S1-S22)


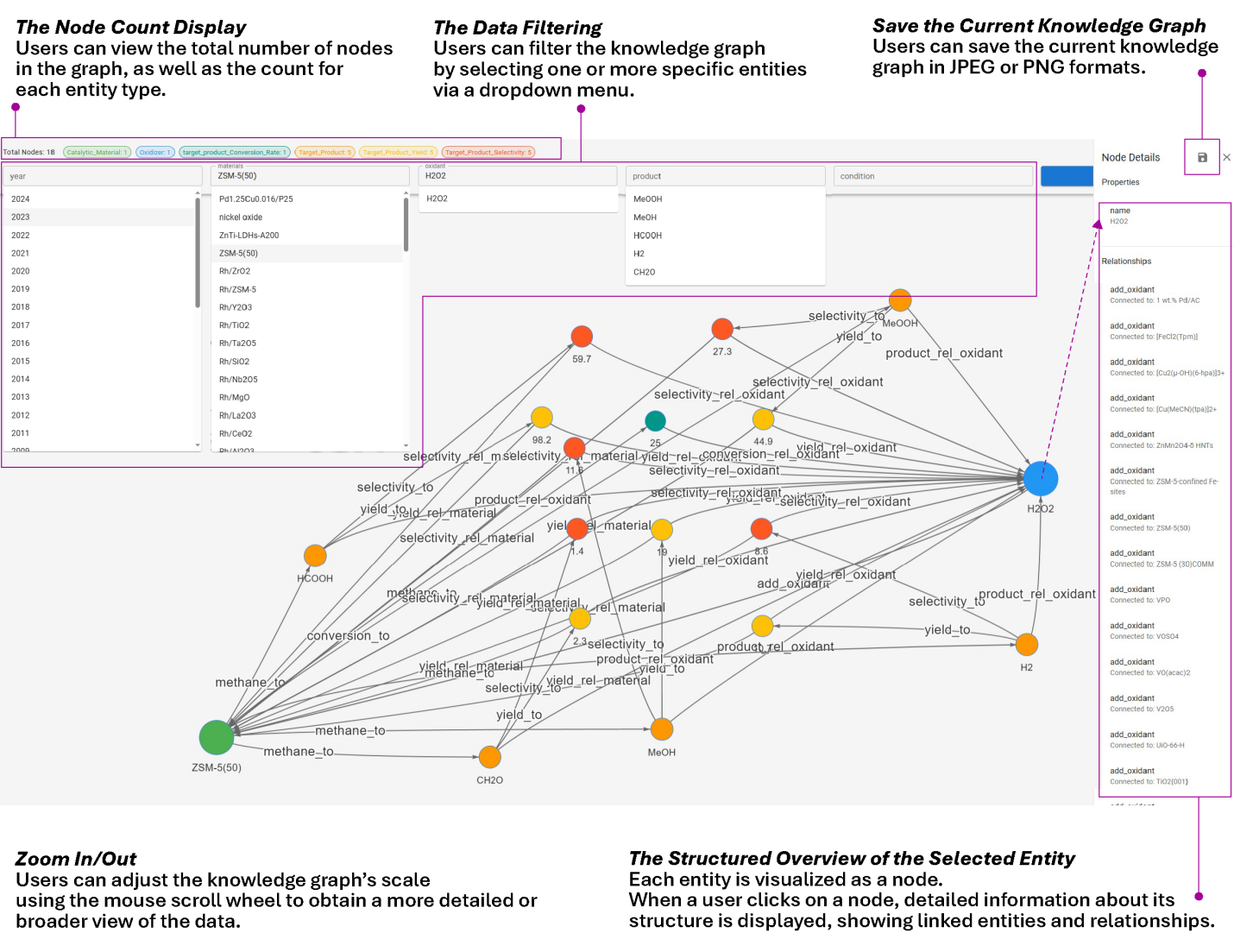


**Figure S1.** A user-friendly, code-free interface supports intuitive querying and visualization of CH_4_-KG (<http://139.224.202.44:3000/>), providing researchers, regardless of AI expertise, with a comprehensive, structured overview of methane selective conversion. For example, the user can select the publication year “2023”, the catalytic material “ZSM-5(50)”, and the oxidizer “H₂O₂” to explore the targeted products and their corresponding yields and selectivities.


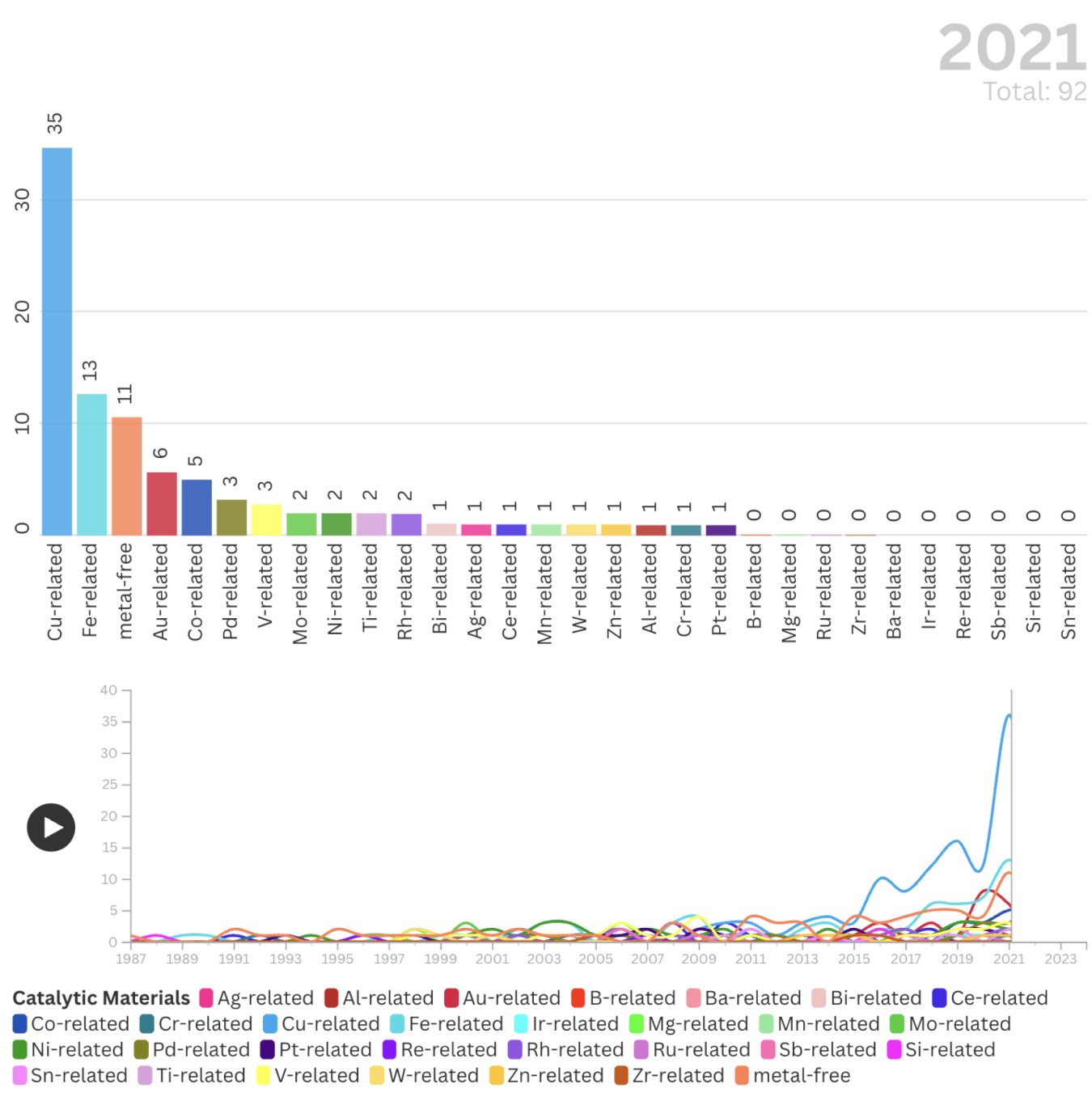


**Figure S2.** Yearly dynamic trends of emerging catalytic material categories since 1987. Users can observe the yearly dynamics of emerging and disappearing catalytic material categories, along with the number of catalytic material-related publications. The bars are arranged from left to right according to the number of catalytic material-related publications, from highest to lowest. At the top right, users can view the total number of publications for each year. They can also drag the timeline at the bottom to explore catalytic material categories for a specific year. The curve illustrates variations in the number of catalytic material-related publications. The yearly dynamic trends of emerging catalytic material categories can be viewed at <https://public.flourish.studio/visualisation/20558903/>.

**
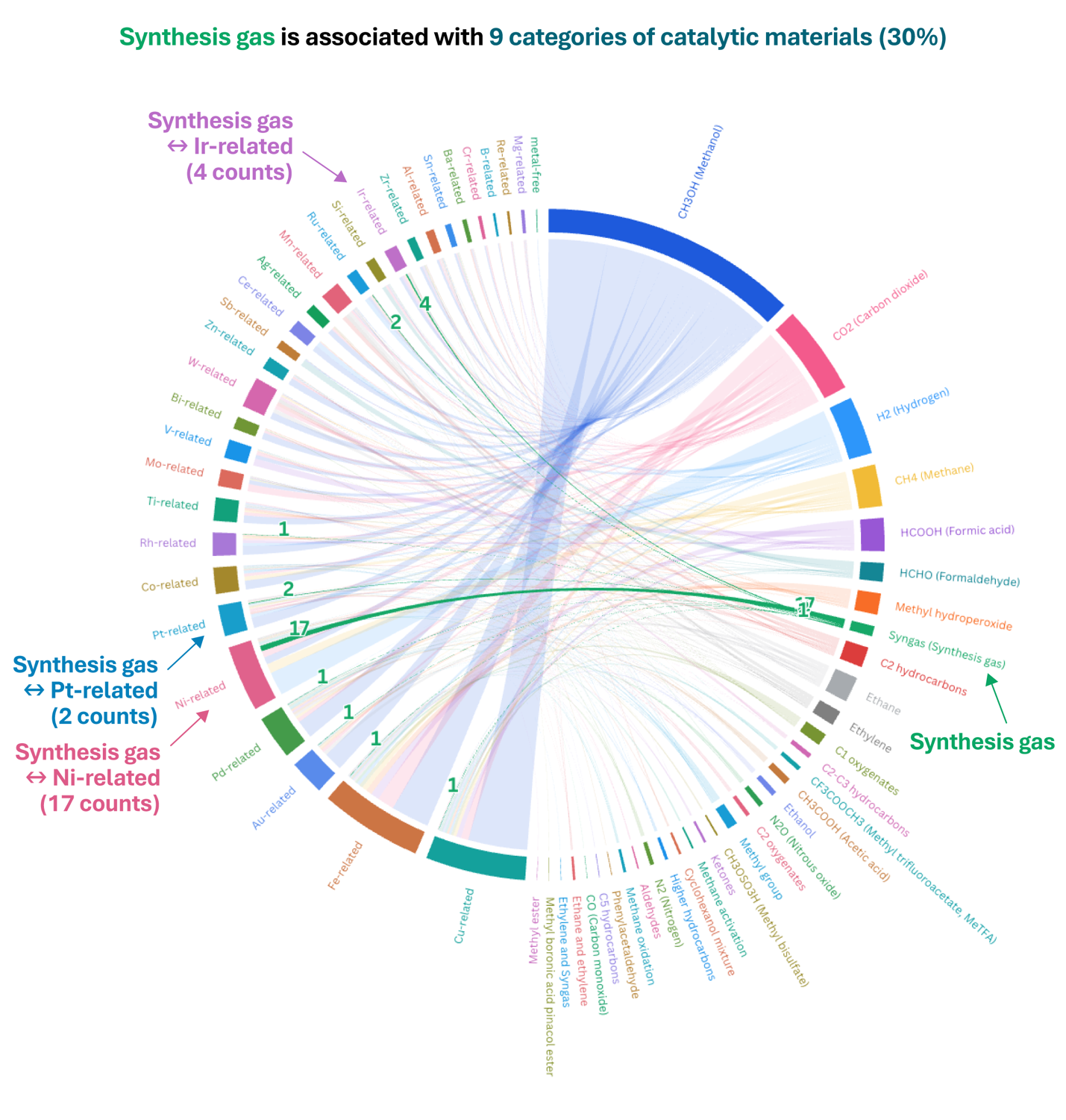
**

**Figure S3.** Synthesis gas is associated with 9 categories of catalytic materials, such as Ir-, Pt-, and Ni-related materials.

<https://public.flourish.studio/visualisation/25051337/>

**
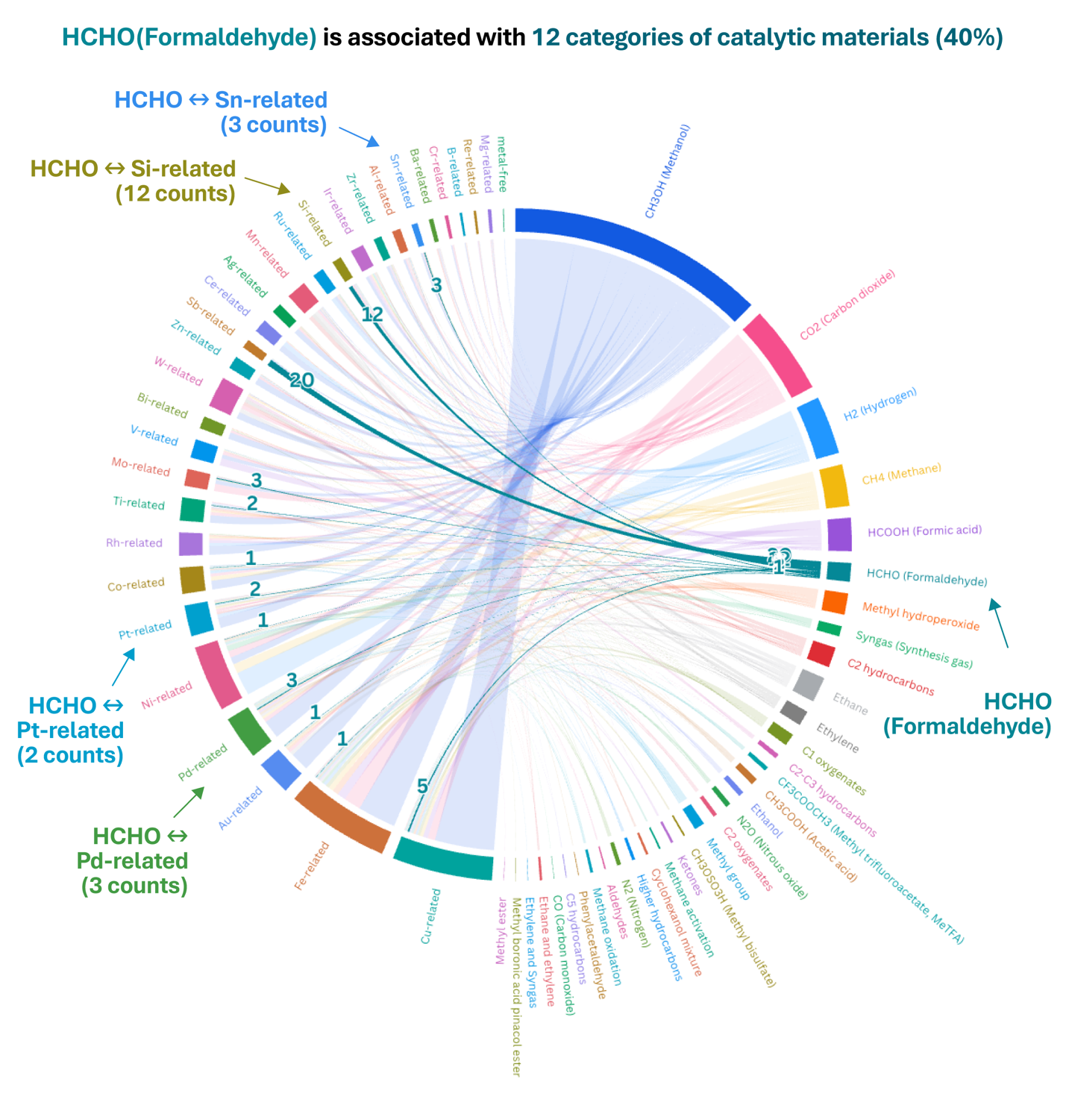
**

**Figure S4.** Formaldehyde is associated with 12 categories of catalytic materials, such as Sn-, Si-, Pt-, and Pd-related materials.

<https://public.flourish.studio/visualisation/25051337/>


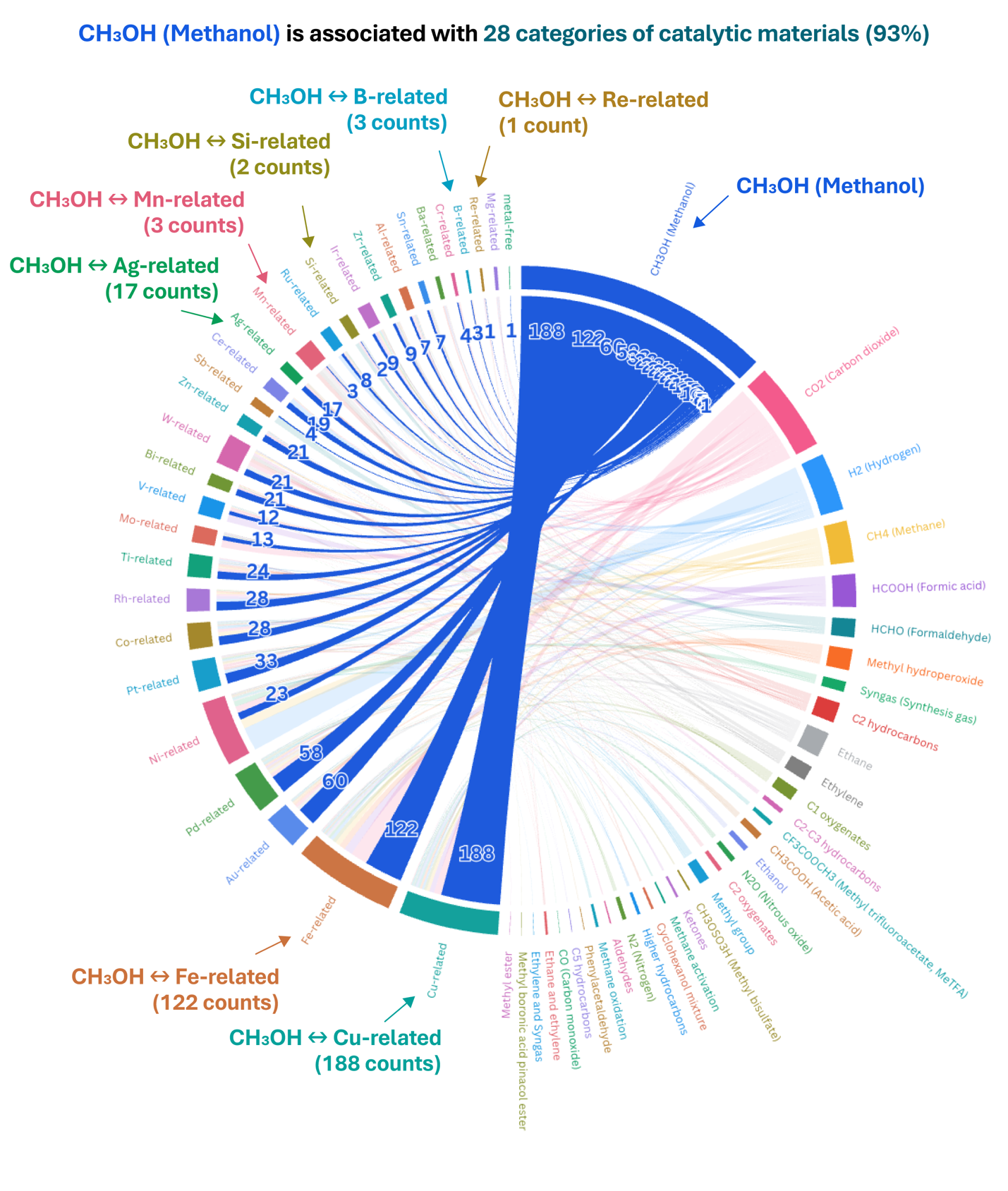


**Figure S5.** Methanol is associated with 28 categories of catalytic materials, such as B-related and Mn-related materials.

<https://public.flourish.studio/visualisation/25051337/>

**
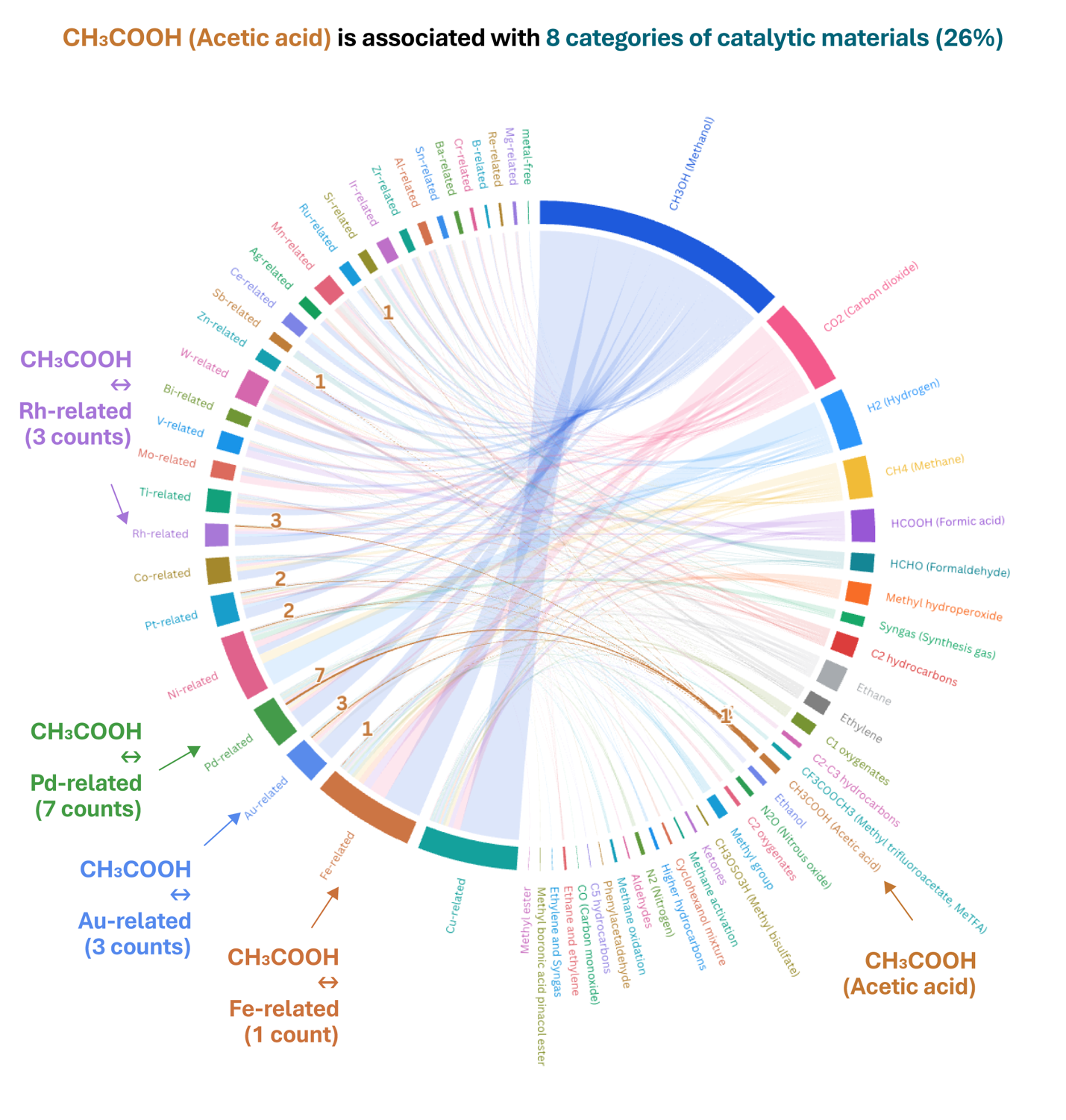
**

**Figure S6.** Acetic acid is associated with 8 categories of catalytic materials, such as Rh-, Pd-, Au-, and Fe-related materials.

<https://public.flourish.studio/visualisation/25051337/>


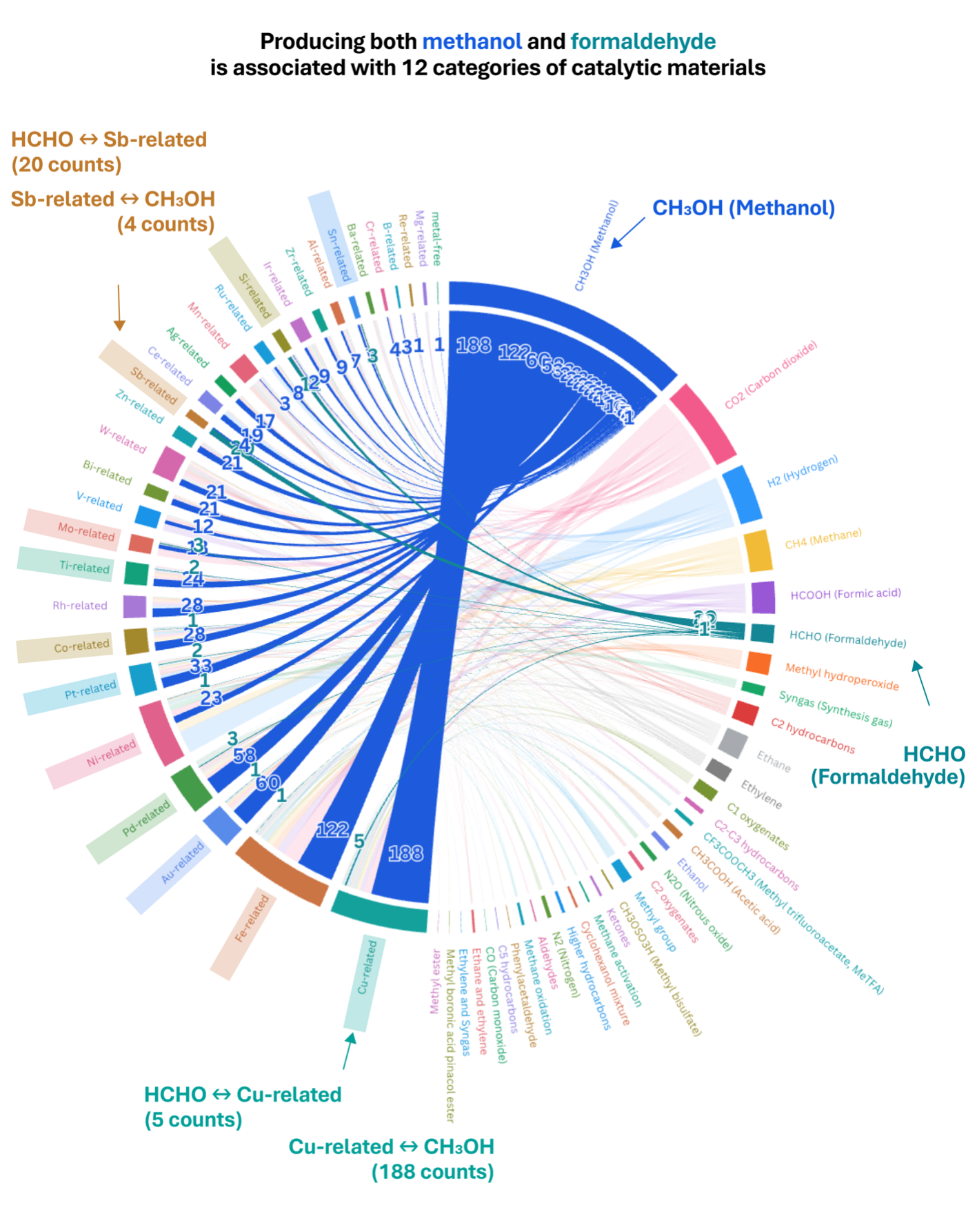


**Figure S7.** Producing both methanol and formaldehyde is associated with 12 categories of catalytic materials, such as Cu-, and Sb-related materials. <https://public.flourish.studio/visualisation/25051337/>

**
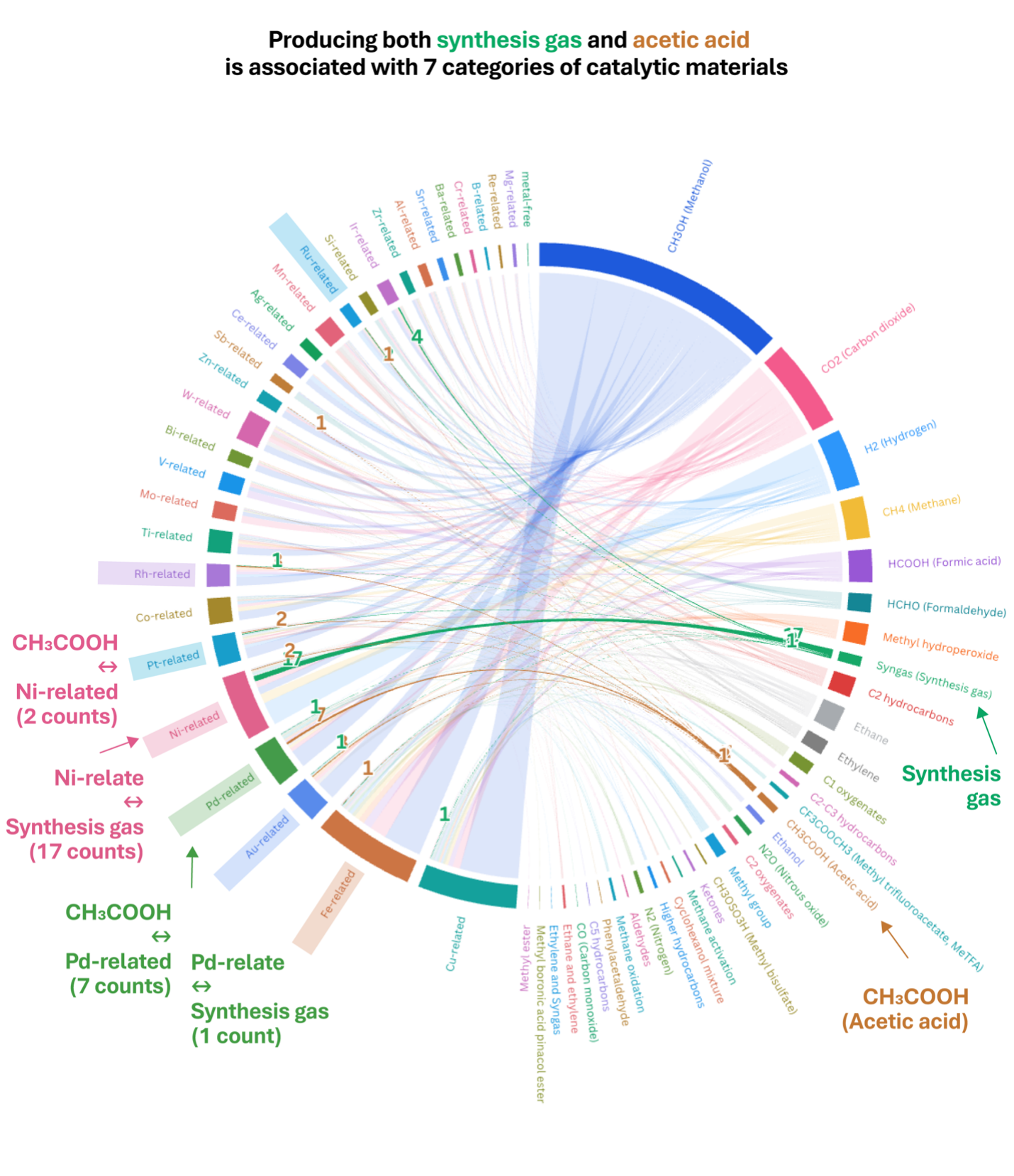
**

**Figure S8.** Producing both synthesis gas and acetic acid is associated with 7 categories of catalytic materials, such as Ni-, and Pd-related materials. <https://public.flourish.studio/visualisation/25051337/>


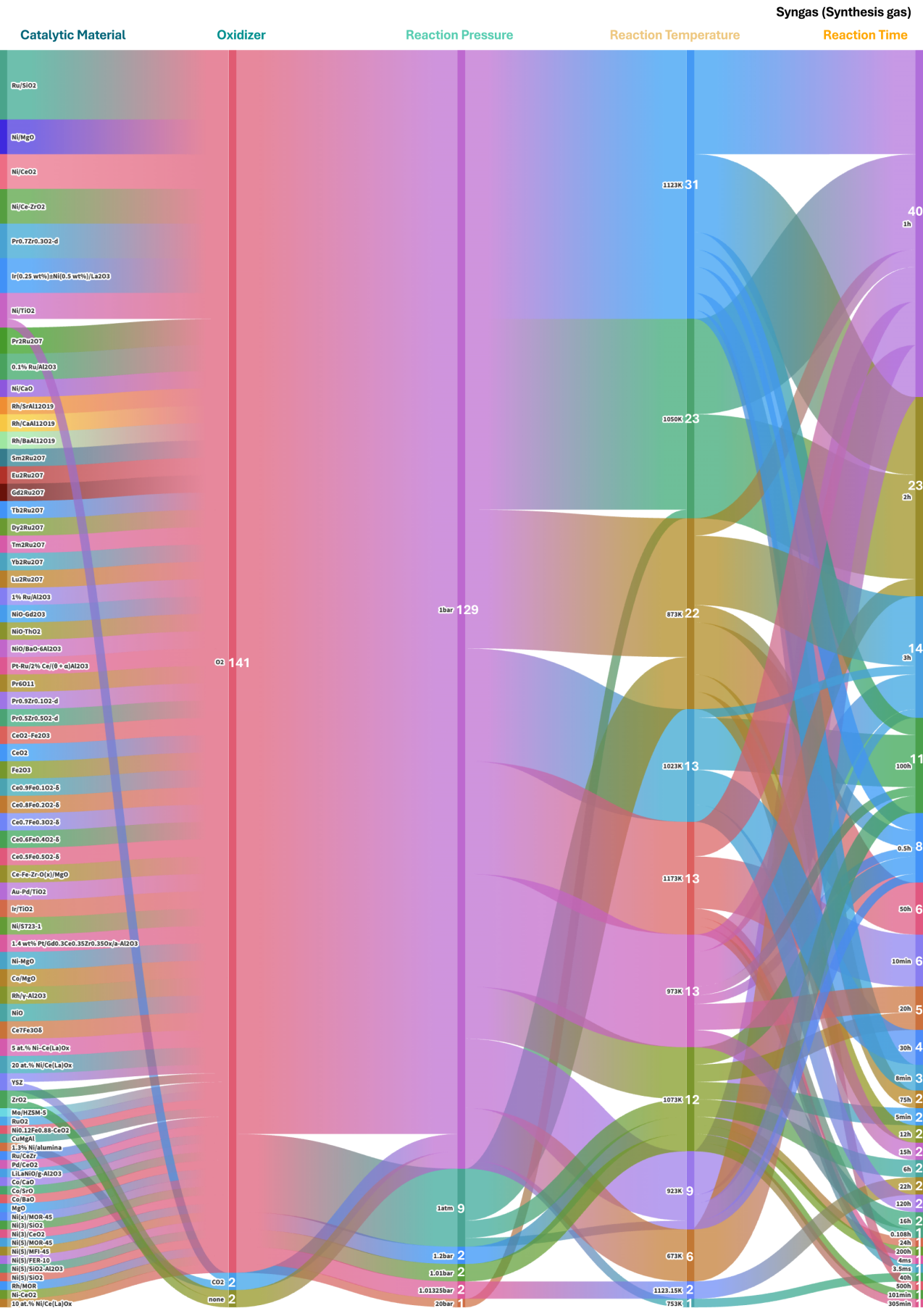


**Figure S9.** The reaction conditions for conversion of methane to synthesis gas. <https://public.flourish.studio/visualisation/22186298/>


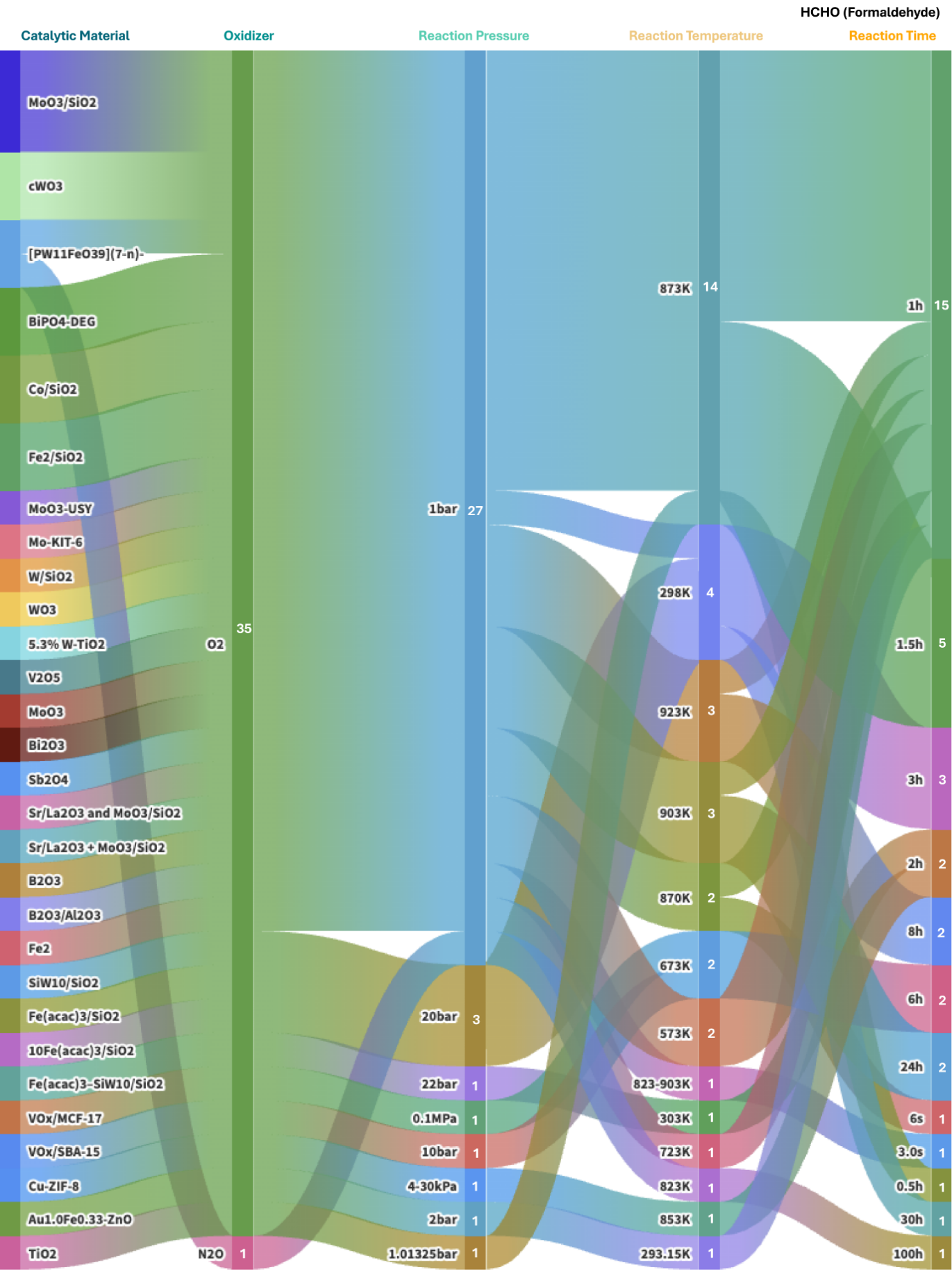


**Figure S10.** The reaction conditions for conversion of methane to formaldehyde. <https://public.flourish.studio/visualisation/22164993/>


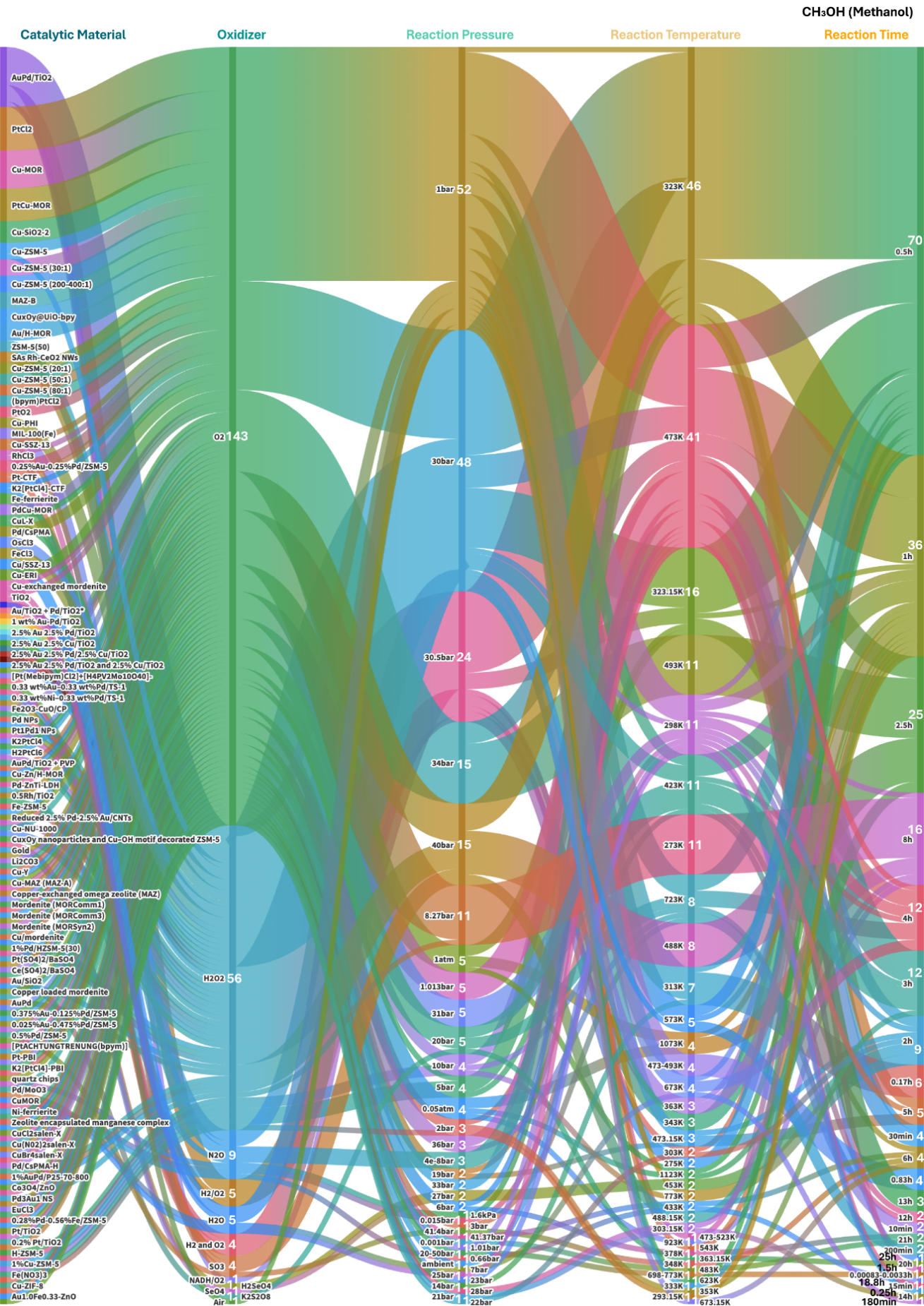


**Figure S11**. The reaction conditions for conversion of methane to methanol. <https://public.flourish.studio/visualisation/22225150/>


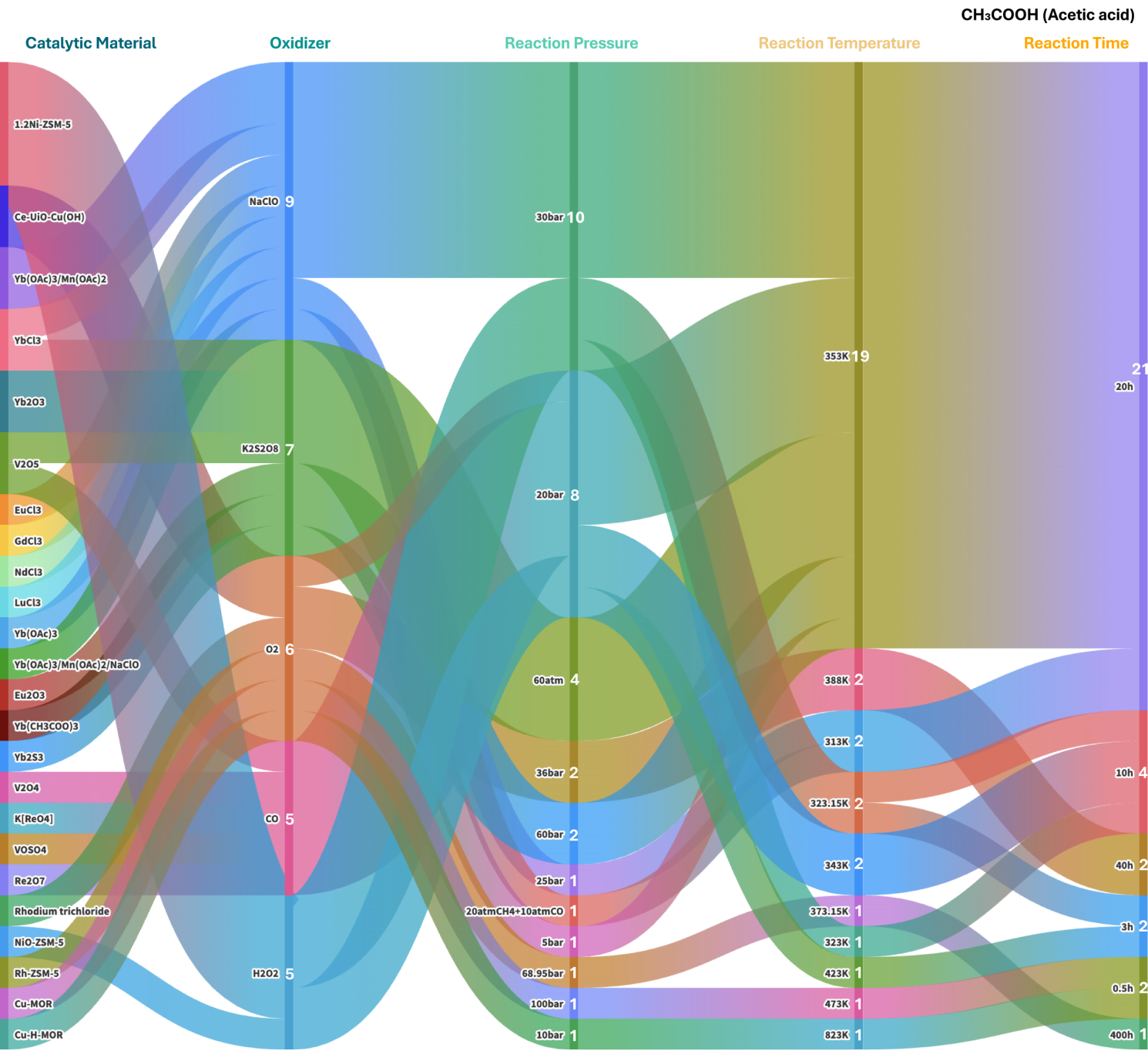


**Figure S12**. The reaction conditions for conversion of methane to acetic acid. <https://public.flourish.studio/visualisation/22223054/>


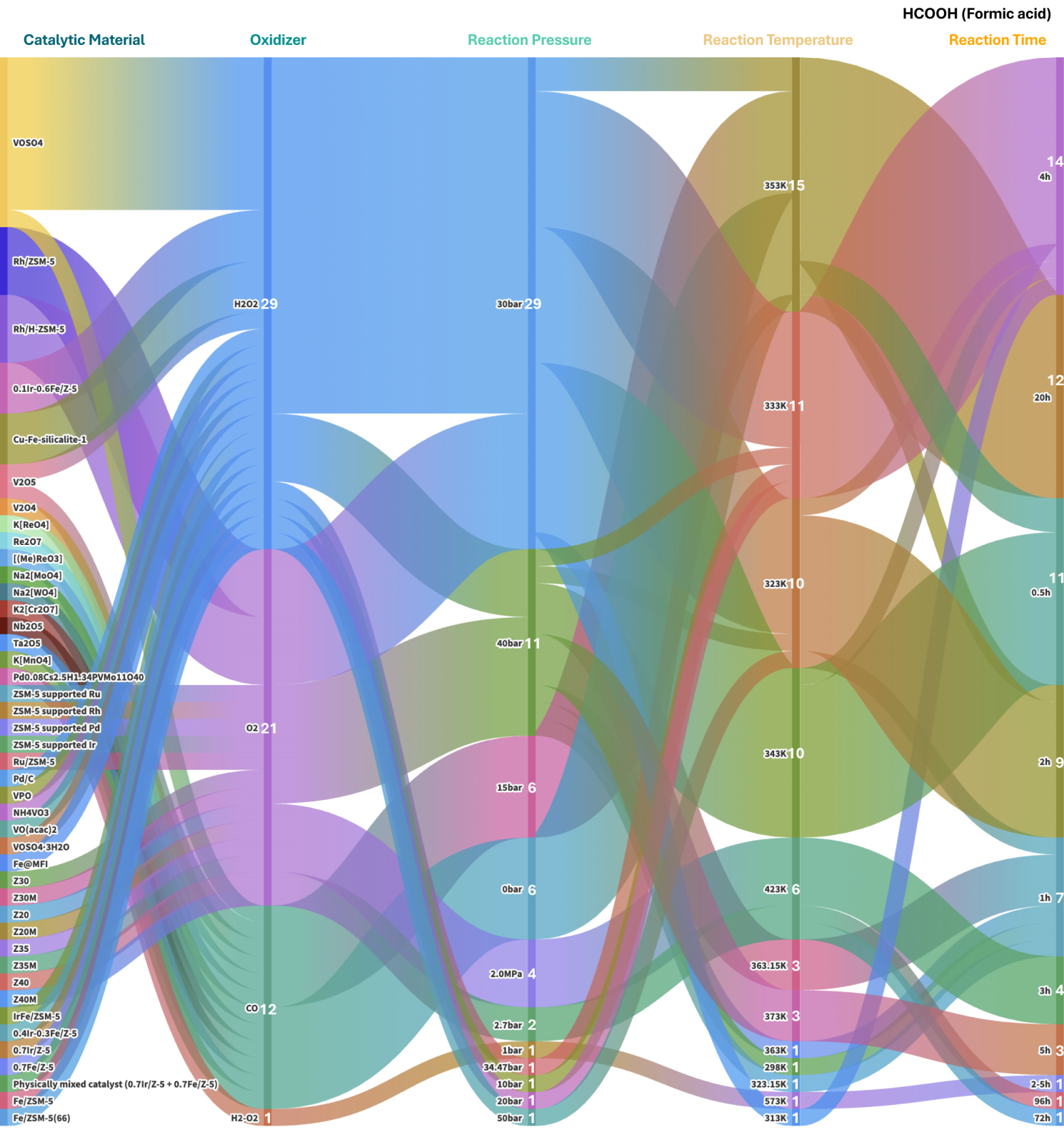


**Figure S13.** The reaction conditions for conversion of methane to formic acid. <https://public.flourish.studio/visualisation/22223437/>


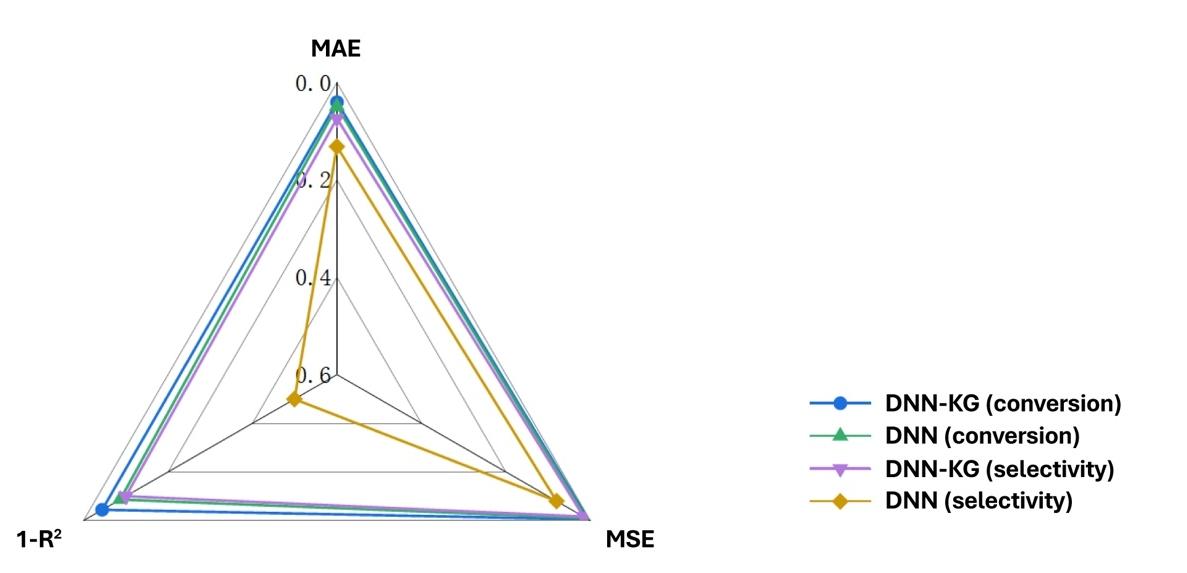
**Figure S14.** Comparison of Model Performance. We compare the performance of traditional methods and our proposed approach in predicting product selectivity and methane conversion. For intuitive visualization, the reciprocals of the mean squared error (MSE) and mean absolute error (MAE) are normalized.

**
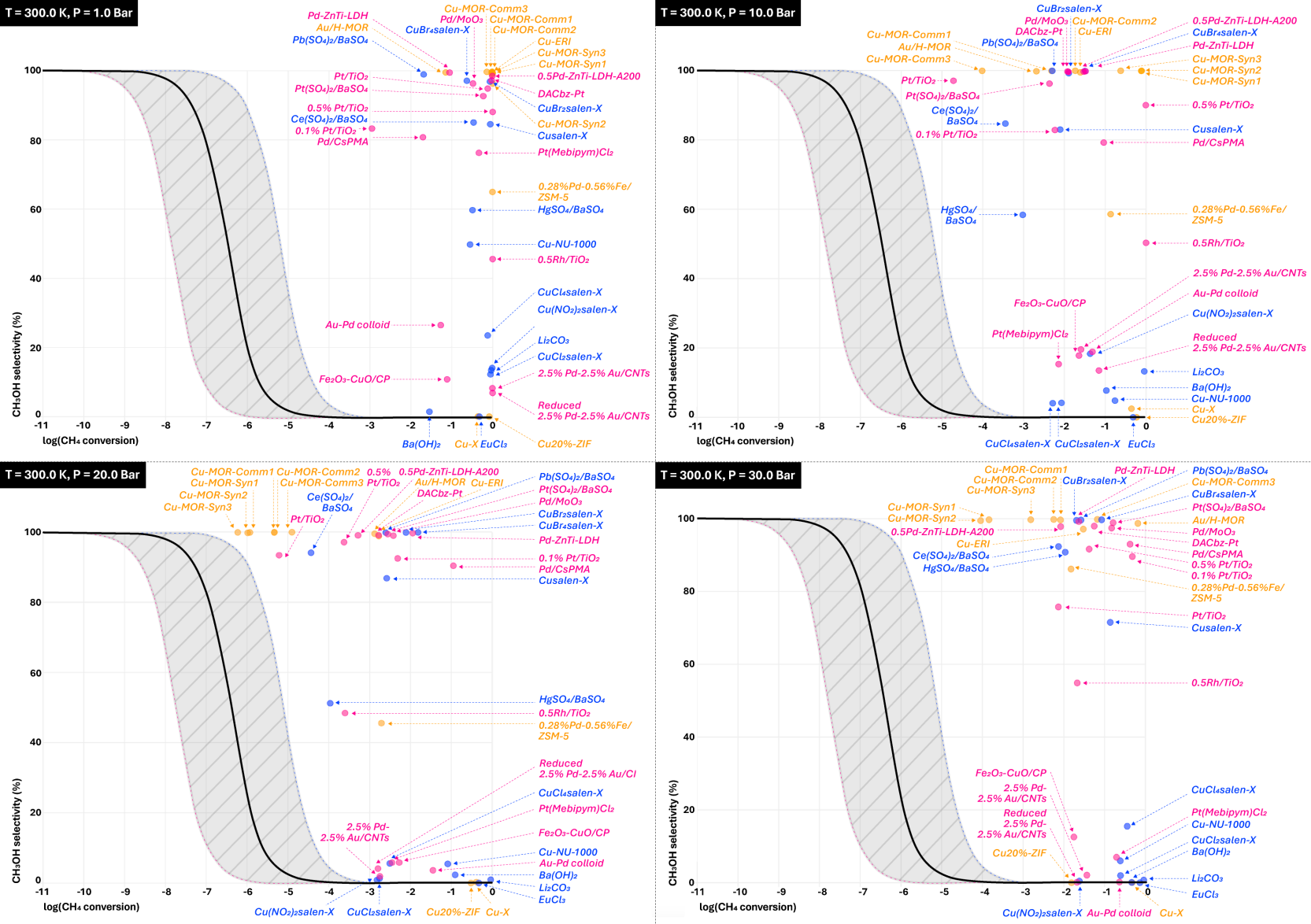
**

**Figure S15**. Predicted CH_3_OH selectivity and CH_4_ conversion at 300.0 K under pressures of 1.0, 10.0, 20.0, or 30.0 bar. Zeolites- and MOFs-based catalysts (orange dots), transition metal- and metal salt-based catalysts (blue dots) and noble metal-based catalysts (pink dots) overlaid on the non-catalytic selectivity-conversion trade-off line described by Equation (1) at 300 K under pressures of 1.0, 10.0, 20.0, or 30.0 bar using ΔG_DFT_ (black line) including a ±1 σ error (the shading area between blue and pink dash lines).

**
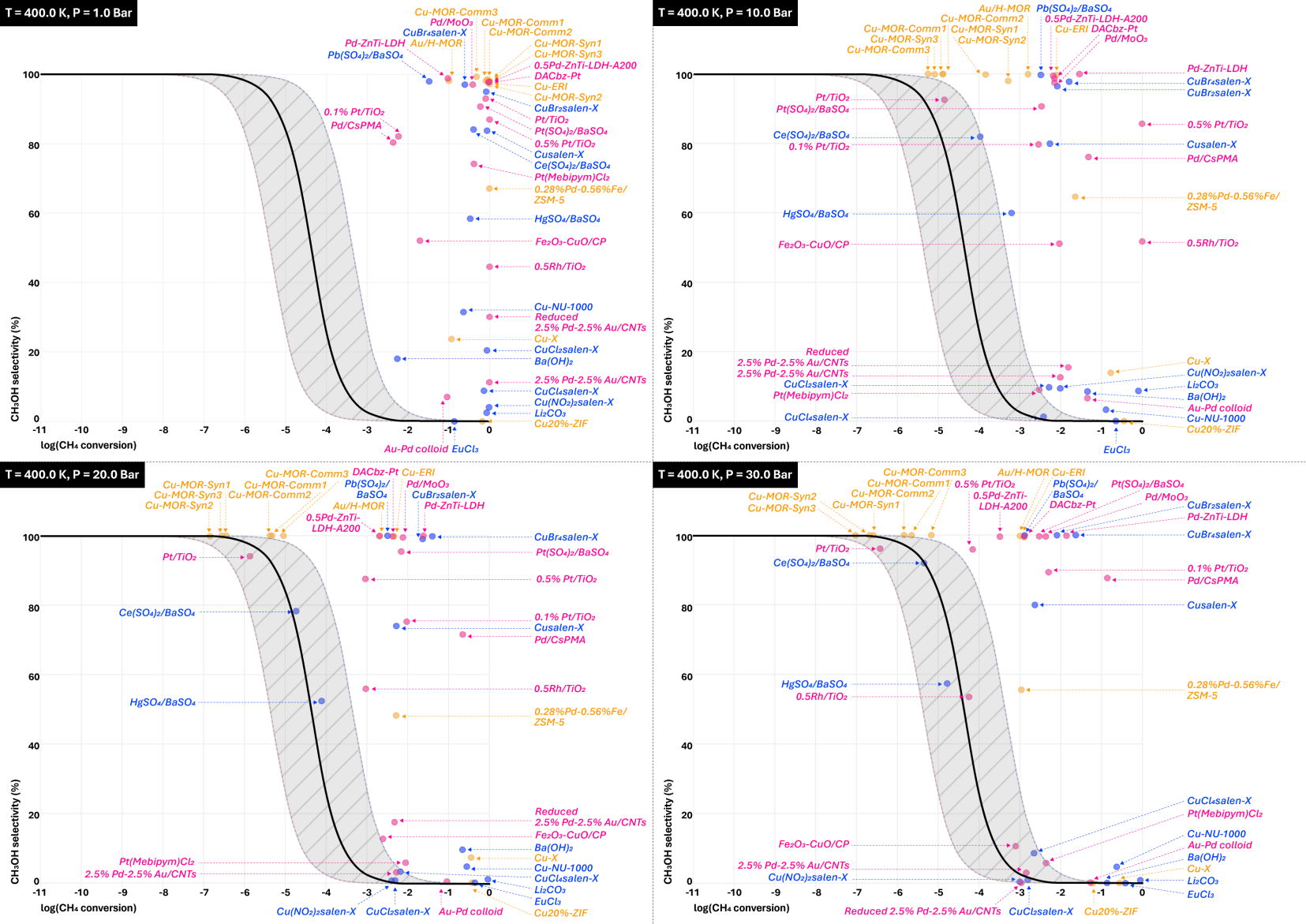
**

**Figure S16**. Predicted CH_3_OH selectivity and CH_4_ conversion at 400.0 K under pressures of 1.0, 10.0, 20.0, or 30.0 bar. Zeolites- and MOFs-based catalysts (orange dots), transition metal- and metal salt-based catalysts (blue dots) and noble metal-based catalysts (pink dots) overlaid on the non-catalytic selectivity-conversion trade-off line described by Equation (1) at 400 K under pressures of 1.0, 10.0, 20.0, or 30.0 bar using ΔG_DFT_ (black line) including a ±1 σ error (the shading area between blue and pink dash lines).


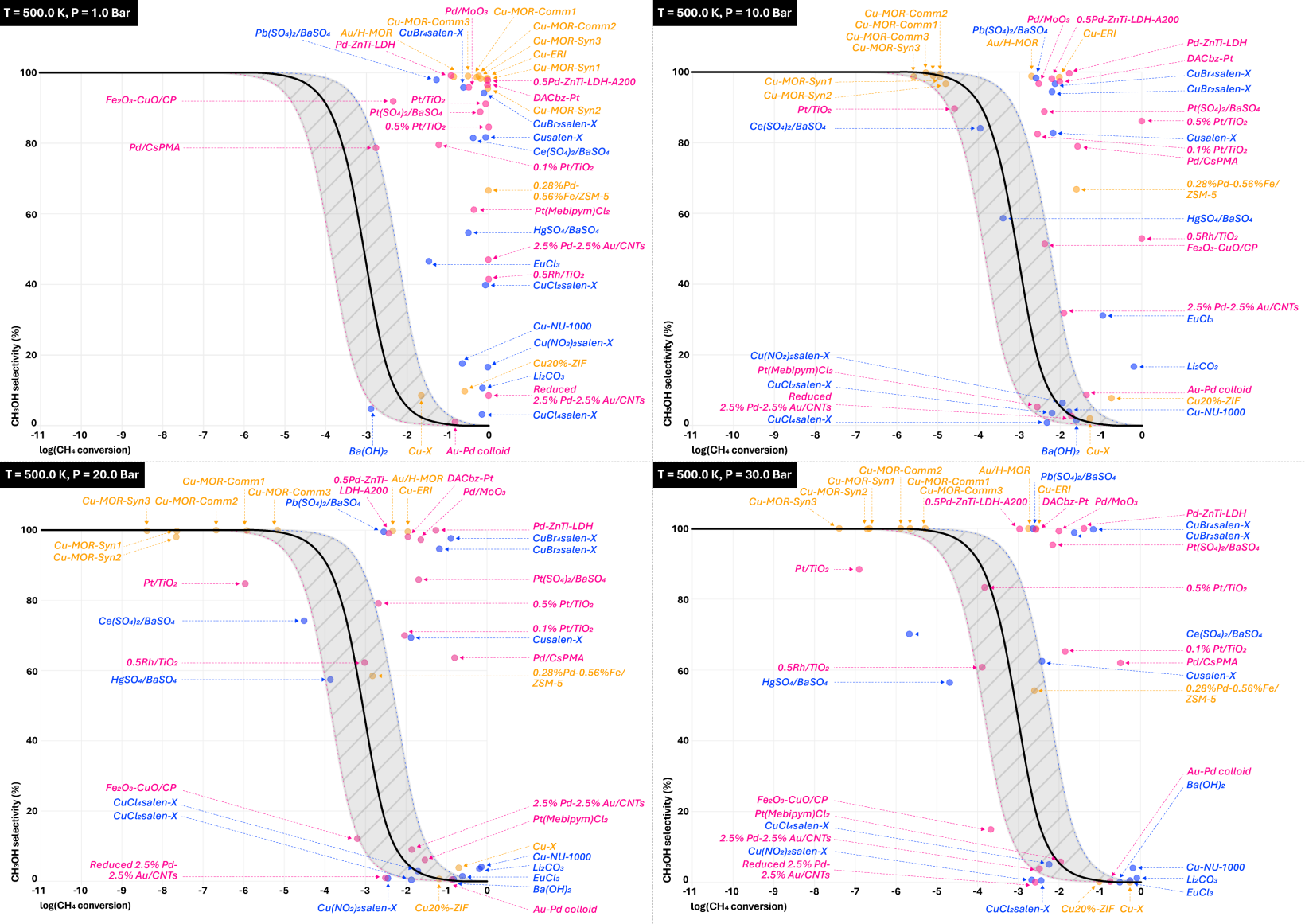


**Figure S17**. Predicted CH_3_OH selectivity and CH_4_ conversion at 500.0 K under pressures of 1.0, 10.0, 20.0, or 30.0 bar. Zeolites- and MOFs-based catalysts (orange dots), transition metal- and metal salt-based catalysts (blue dots) and noble metal-based catalysts (pink dots) overlaid on the non-catalytic selectivity-conversion trade-off line described by Equation (1) at 500 K under pressures of 1.0, 10.0, 20.0, or 30.0 bar using ΔG_DFT_ (black line) including a ±1 σ error (the shading area between blue and pink dash lines).


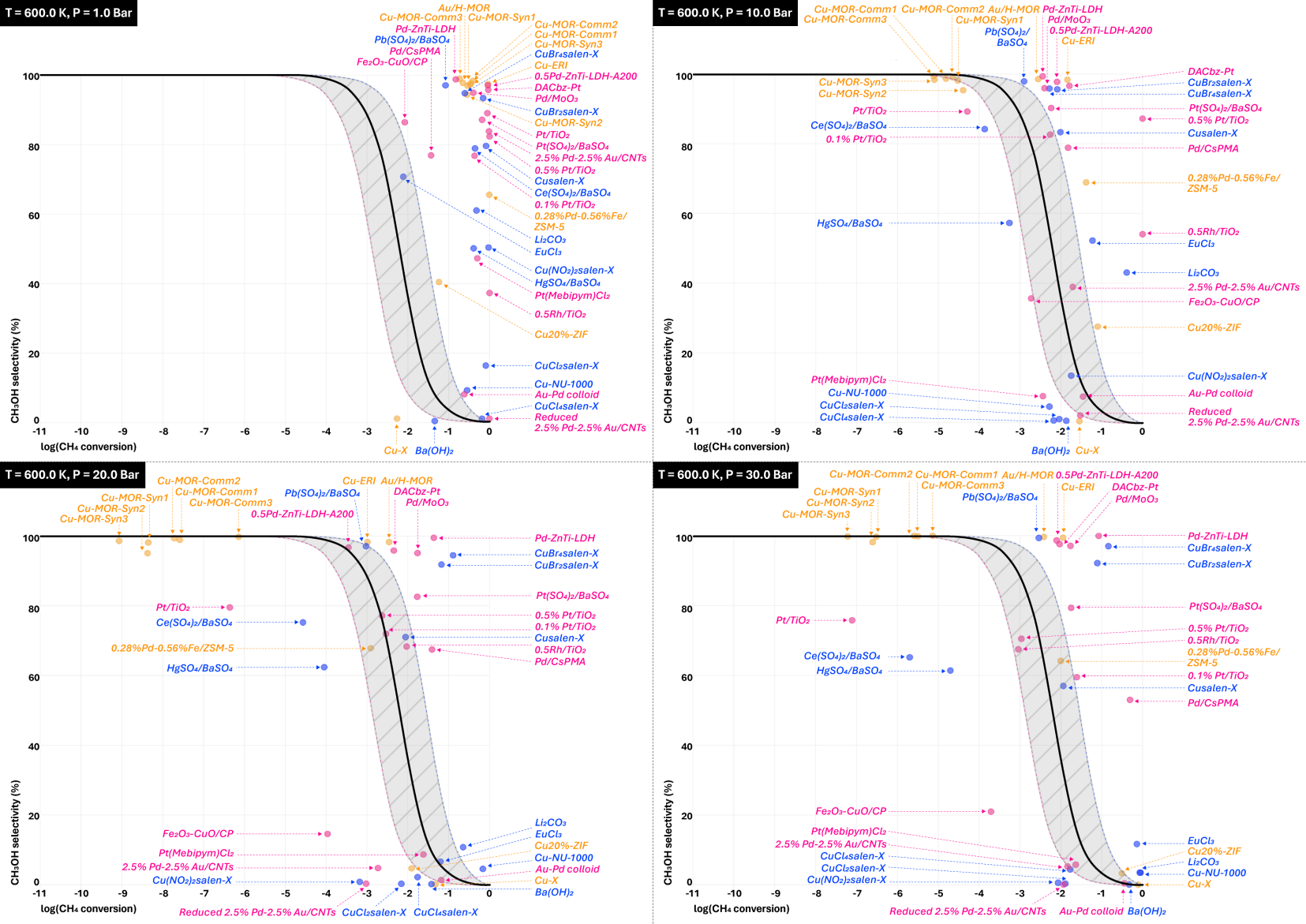


**Figure S18**. Predicted CH_3_OH selectivity and CH_4_ conversion at 600.0 K under pressures of 1.0, 10.0, 20.0, or 30.0 bar. Zeolites- and MOFs-based catalysts (orange dots), transition metal- and metal salt-based catalysts (blue dots) and noble metal-based catalysts (pink dots) overlaid on the non-catalytic selectivity-conversion trade-off line described by Equation (1) at 600 K under pressures of 1.0, 10.0, 20.0, or 30.0 bar using ΔG_DFT_ (black line) including a ±1 σ error (the shading area between blue and pink dash lines).


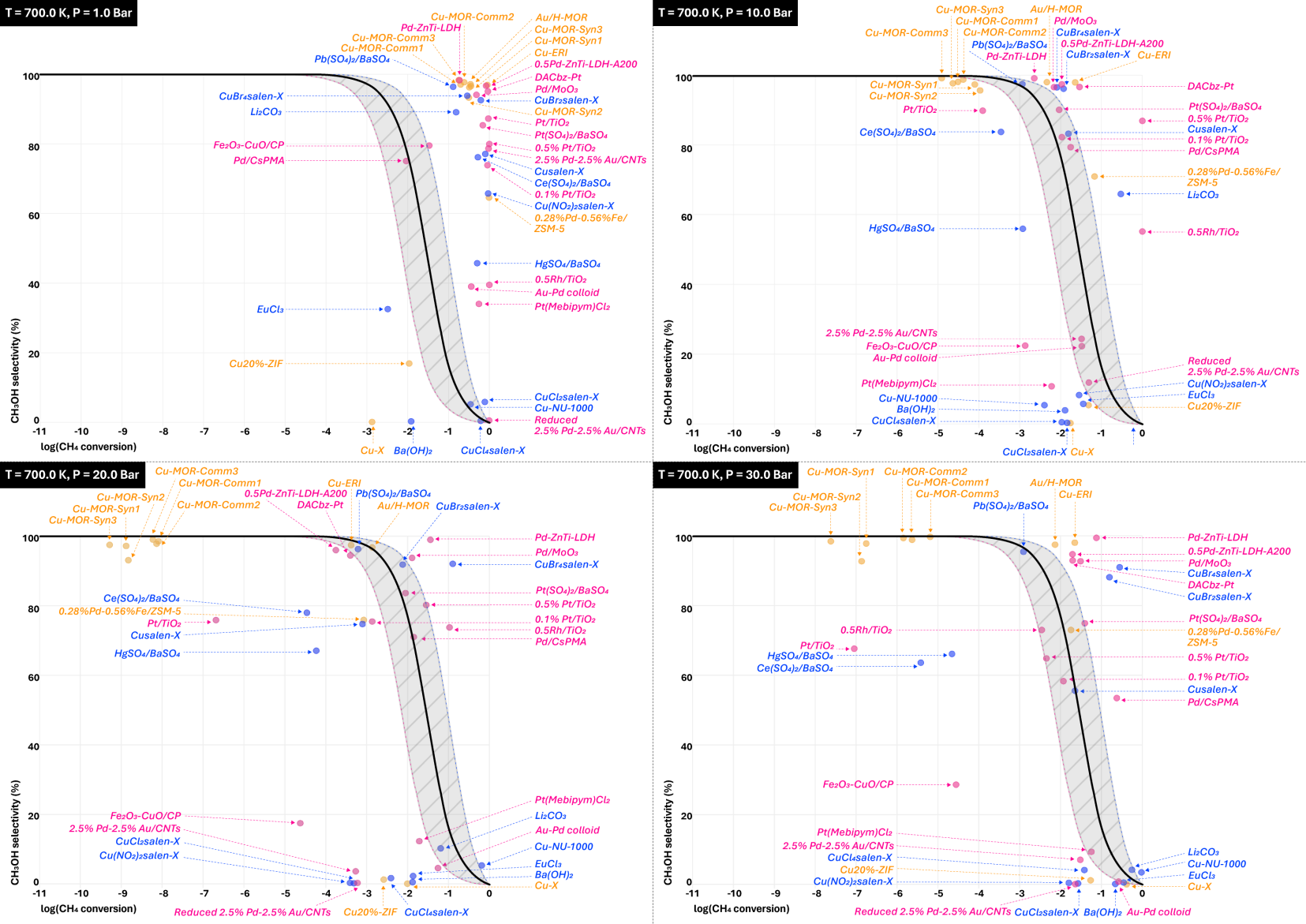


**Figure S19**. Predicted CH_3_OH selectivity and CH_4_ conversion at 700.0 K under pressures of 1.0, 10.0, 20.0, or 30.0 bar. Zeolites- and MOFs-based catalysts (orange dots), transition metal- and metal salt-based catalysts (blue dots) and noble metal-based catalysts (pink dots) overlaid on the non-catalytic selectivity-conversion trade-off line described by Equation (1) at 700 K under pressures of 1.0, 10.0, 20.0, or 30.0 bar using ΔG_DFT_ (black line) including a ±1 σ error (the shading area between blue and pink dash lines).


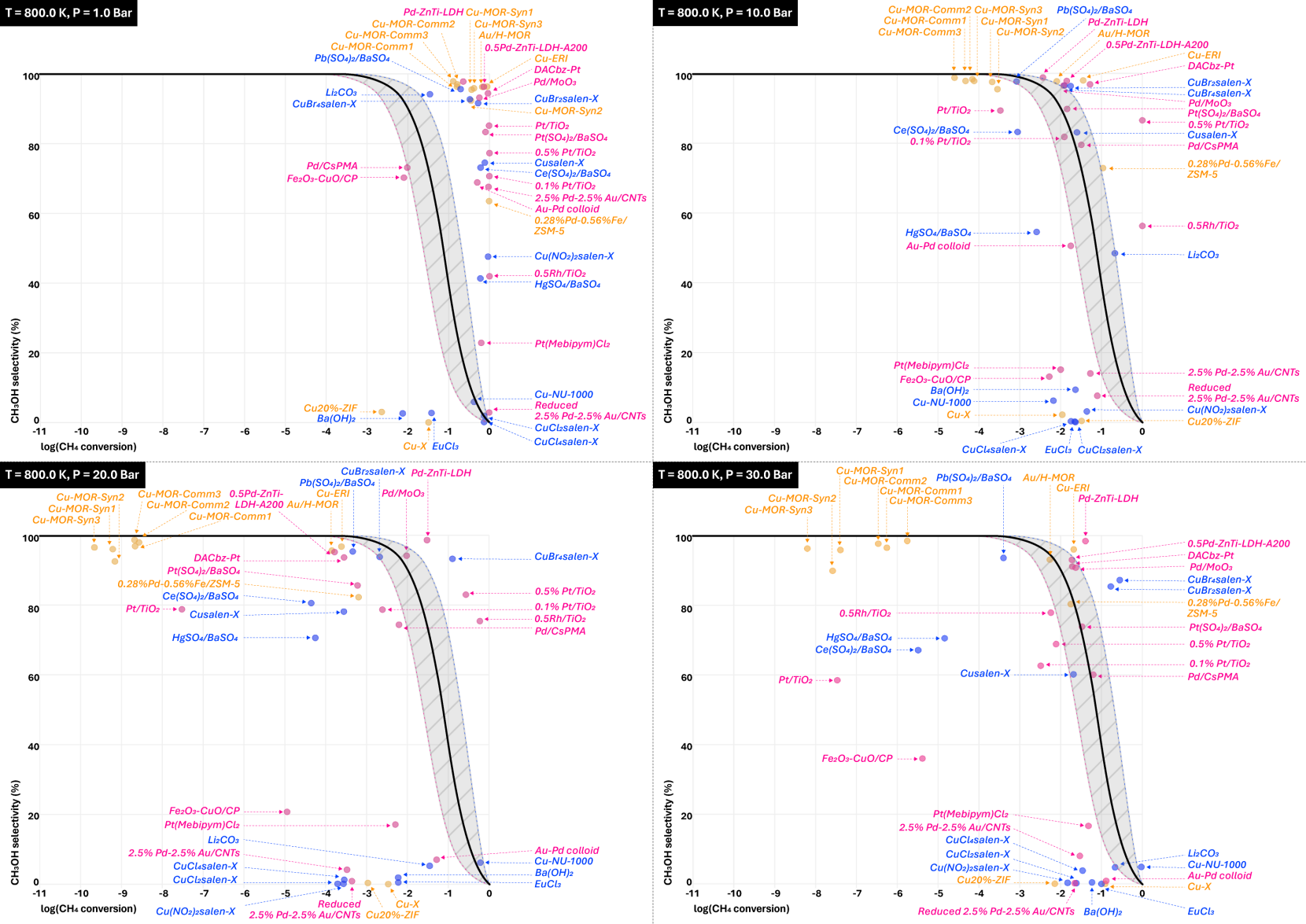


**Figure S20**. Predicted CH_3_OH selectivity and CH_4_ conversion at 800.0 K under pressures of 1.0, 10.0, 20.0, or 30.0 bar. Zeolites- and MOFs-based catalysts (orange dots), transition metal- and metal salt-based catalysts (blue dots) and noble metal-based catalysts (pink dots) overlaid on the non-catalytic selectivity-conversion trade-off line described by Equation (1) at 800 K under pressures of 1.0, 10.0, 20.0, or 30.0 bar using ΔG_DFT_ (black line) including a ±1 σ error (the shading area between blue and pink dash lines).

**
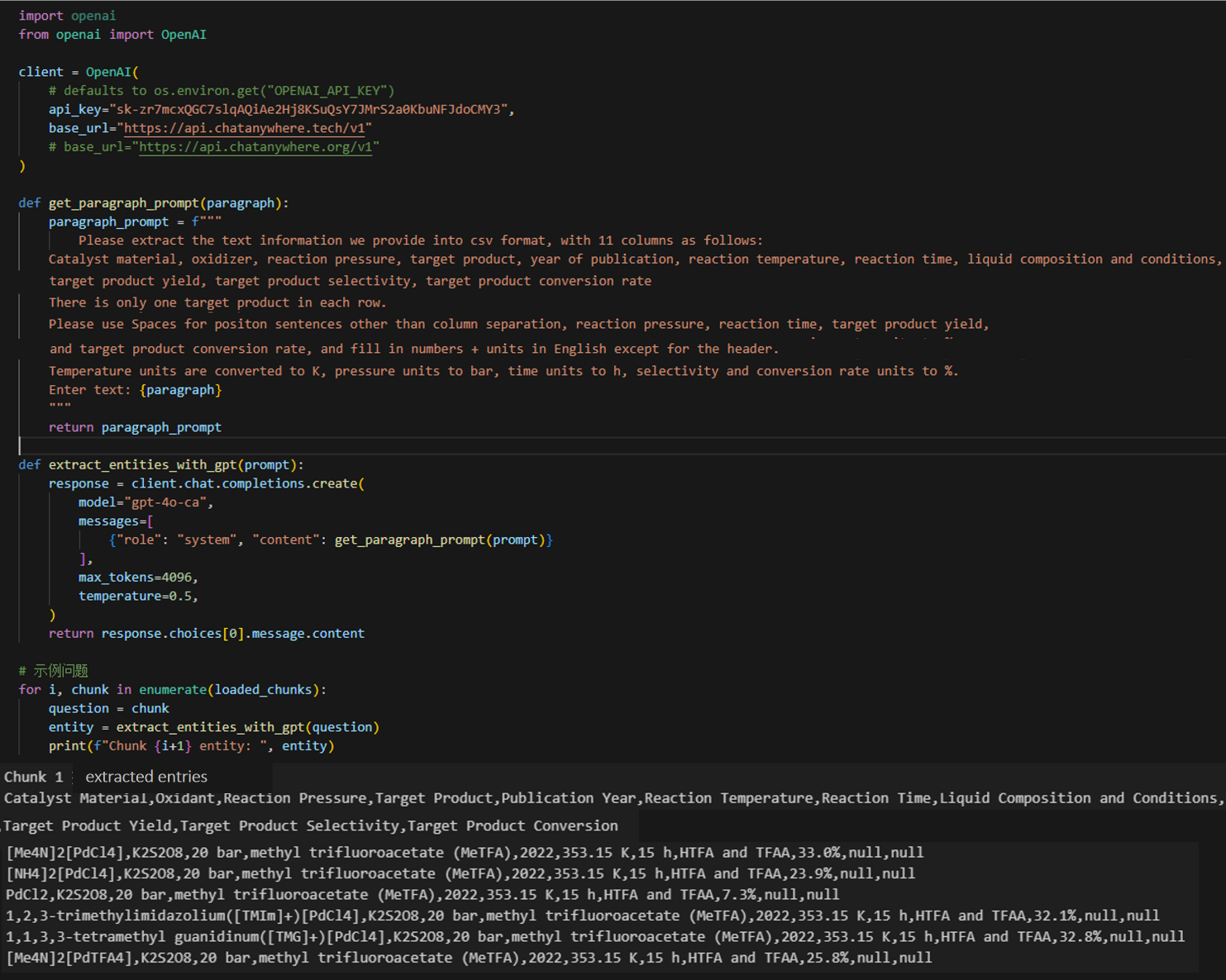
**

**Figure S21.** Illustrates an example of entity extraction using GPT-4o-ca through prompts.
*“Please extract the text information we provide into CSV format with 11 columns as follows Catalytic Material, Oxidizer, Reaction Pressure, Target Product, Published Year, Reaction Temperature, Reaction Time, Liquid Composition and Condition, Target Product Yield, Target Product Selectivity, Target Product Conversion Rate. There is only one target product in each row.*
*Please use Spaces for positioning sentences other than column separation, reaction pressure, reaction time, target product yield, target product selectivity, target product conversion rate, and fill in numbers + units in English except for the header. Temperature units are converted to K, pressure units to bar, time units to hours (h), and selectivity and conversion units to %.”*Entity extraction results, for example:
Catalytic Material: 1,2,3-trimethyl imidazolium ([TMIn]+)[PdC_4_]
Reaction Pressure: 20 bar
Target Product: Methyl trifluoroacetate (MeTFA).


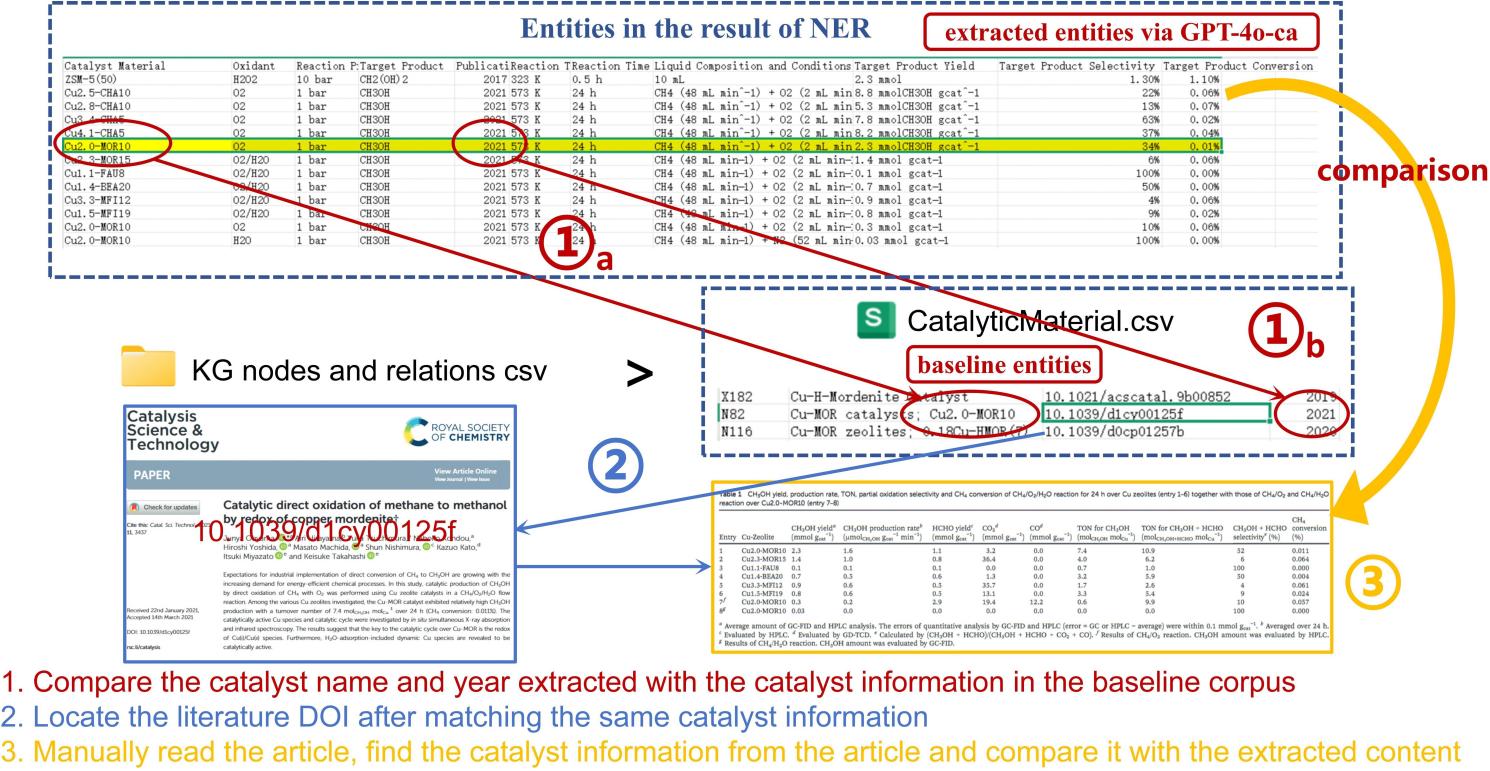


**Figure S22.** Illustrates the accuracy of entities extracted by GPT-4o-ca. i) We can identify a specific catalytic material, such as Cu2.0-MOR10, in the automatically extracted entities (①_a_) and determine the publication year in which it was mentioned; ii) We locate the same catalytic material and corresponding publication year in the baseline entities (①_b_) to compare the results with those found in the automatically extracted entities (①_a_); iii) We then retrieve the corresponding DOI link (②) and access the full paper that mentions this catalytic material; iv) We review the paper (③) to verify whether the automatically extracted entities are accurate and useful.

3. Supporting Tables (Table S1-S6)

**Table S1.** The comparison of the predicted and reported methane conversion and methanol selectivity for five randomly selected catalysts.

| **Catalysts** | **Reaction conditions** | | | **Conversion (%)**  **Reported/Predicted** | **Selectivity (%)**  **Reported/Predicted** |
| --- | --- | --- | --- | --- | --- |
|  | **Pressure (bar)** | **Temp (K)** | **Time (h)** |  |  |
| **Cu-ERI^[1]^** | 30 | 573 | 0.5 | Non/0.62 | 90.4/87 |
| **Pd-ZnTi-LDH^[2]^** | 20 | 298 | 10 | Non/0.81 | 99.7/100 |
| **BiPO_4_-DEG^[3]^** | 1 | 823 | 24 | 0.55 ± 0.02/0.50 | 60.6 ± 1.6/60.03 |
| **V/Ti-SBA-15^[4]^** | 1 | 823 | 1 | 0.25/0.47 | 75.41/75 |
| **Pt-Ru Al_2_O_3_^[5]^** | 1 | 1173 | 0.00015 | 96.70/96~100 | 100/99.50 |

**Table S2.** Eleven entity types relevant to methane selective conversion experiments are identified, along with their descriptions and examples.

| Entity type | Description | Examples |
| --- | --- | --- |
| Published Year | The year when the article was published | 1986, 2000, 2016 |
| Catalytic Material | The catalytic material(s) used in methane selective conversion | CeO_2_/Cu_2_O/Cu(111),  0.28 %Pd-0.56 %Fe/ZSM-5,  5% Pd/C |
| Oxidizer | The substance that provides oxygen or oxygen-containing compounds during methane selective conversion | O_2_, H_2_O_2_, water |
| Reaction Pressure | The pressure applied during methane selective conversion | 20 bar, room pressure, atmospheric pressure |
| Reaction Temperature | The temperature maintained during methane selective conversion | 500 K, >623 K, 773-963 K, |
| Reaction Time | The duration of the methane selective conversion reaction | between 1 and 8 h, from 4 to 20 h, 2h |
| Liquid Composition and Condition | The liquid phase used or present during methane selective conversion | concentrated sulfuric acid, in pure water, in the steam |
| Target Product | The compound that is produced as the target product during methane selective conversion | methanol, acetic acid, C1 oxygenate products (CH_3_OH, CH_3_OOH, and HCHO) |
| Target Product Yield | The amount of the target product that is obtained from methane selective conversion | methanol yield of 16.4%, formaldehyde yield up to 3%, 10% of the combined yield of CH_3_OH and HCHO |
| Target Product Selectivity | The proportion of the target product relative to the total products that are formed during methane selective conversion | methanol selectivity of up to 83%, formic acid selectivity up to 81-82%, CH_3_OOH and CH_3_OH with 95% selectivity |
| Target Product Conversion Rate | The percentage of methane that is converted into the target product during methane selective conversion | a methane conversion of 1-3 % over Pt/TiO_2_ |

**Table S3.** Thirty-two ttypes of relationships between two entities (see **Table S2**) and their corresponding descriptions.

| Relationship type | Description |
| --- | --- |
| “Catalytic Material” to “Published Year”: appear_in | The publication year of an article involving catalytic material(s) |
| “Catalytic Material” to “Target Product Conversion Rate”: conversion_to | The methane conversion achieved when using the catalytic material during methane selective conversion |
| “Catalytic Material” to “Target Product”: methane_to | The conversion of methane into a specific product using a catalytic material |
| “Target Product” to “Target Product Selectivity”: selectivity_to | The selectivity for a particular product during methane selective conversion |
| “Target Product Selectivity” to “Catalytic Material”: selectivity_rel_material | The relationship between the selectivity of a product and the catalytic material used |
| “Target Product” to “Target Product Yield”: yield_to | The yield of a specific product obtained from methane selective conversion |
| “Target Product Yield” to “Catalytic Material”: yield_rel_material | The relationship between the yield of a product and the catalytic material used |
| “Oxidizer” to “Catalytic Material”: add_oxidant | The addition of oxidant(s) to catalytic material(s) during methane selective conversion |
| “Reaction Temperature” to “Catalytic Material”: reaction_temperature | The temperature maintained when using the catalytic material during methane selective conversion |
| “Reaction Pressure” to “Catalytic Material”: reaction_pressure | The pressure applied when using the catalytic material during methane selective conversion |
| “Reaction Time” to “Catalytic Material”: reaction_time | The duration when using the catalytic material during methane selective conversion |
| “Liquid Composition and Condition” to “Catalytic Material”: reaction_liquid | The liquid phase used when using the catalytic material during methane selective conversion |
| “Target Product” to “Oxidizer”: product_rel_oxidant | The relationship between a product and the oxidant used |
| “Target Product” to “Reaction Pressure”: product_rel_pressure | The relationship between a product and the pressure applied |
| “Target Product” to “Reaction Temperature”: product_rel_temperature | The relationship between a product and the temperature maintained |
| “Target Product” to “Reaction Time”: product_rel_time | The relationship between a product and the duration of the reaction |
| “Target Product” to “Liquid Composition and Condition”: product_rel_liquid | The relationship between a product and the liquid phase used |
| “Target Product Selectivity” to “Oxidizer”: selectivity_rel_oxidant | The relationship between the product selectivity and the oxidant(s) used |
| “Target Product Selectivity” to “Reaction Pressure”: selectivity_rel_pressure | The relationship between the product selectivity and the pressure applied |
| “Target Product Selectivity” to “Reaction Temperature”: selectivity_rel_temperature | The relationship between the product selectivity and the temperature maintained |
| “Target Product Selectivity” to “Reaction Time”: selectivity_rel_time | The relationship between the product selectivity and the duration of the reaction |
| “Target Product Selectivity” to “Liquid Composition and Condition”: selectivity_rel_liquid | The relationship between the product selectivity and the liquid phase used |
| “Target Product Yield” to “Oxidizer”: yield_rel_oxidant | The relationship between the product yield and the oxidant used |
| “Target Product Yield” to “Reaction Pressure”: yield_rel_pressure | The relationship between the product yield and the pressure applied |
| “Target Product Yield” to “Reaction Temperature”: yield_rel_temperature | The relationship between the product yield and the temperature maintained |
| “Target Product Yield” to “Reaction Time”: yield_rel_time | The relationship between the product yield and the duration of the reaction |
| “Target Product Yield” to “Liquid Composition and Condition”: yield_rel_liquid | The relationship between the product yield and the liquid phase used |
| “Target Product Conversion Rate” to “Oxidizer”: conversion_rel_oxidant | The relationship between the methane conversion and the oxidant used |
| “Target Product Conversion Rate” to “Reaction Pressure”: conversion_rel_pressure | The relationship between the methane conversion and the pressure applied |
| “Target Product Conversion Rate” to “Reaction Temperature”: conversion_rel_temperature | The relationship between the methane conversion and the temperature maintained |
| “Target Product Conversion Rate” to “Reaction Time”: conversion_rel_time | The relationship between the methane conversion and the duration of the reaction |
| “Target Product Conversion Rate” to “Liquid Composition and Condition”: conversion_rel_liquid | The relationship between the methane conversion and the liquid phase used |

**Table S4.** Baseline entity tag count.

| Entity tag | Count |
| --- | --- |
| Published Year | 28 |
| Catalytic Material | 157 |
| Oxidizer | 50 |
| Reaction Pressure | 19 |
| Reaction Temperature | 44 |
| Reaction Time | 6 |
| Liquid Composition and Condition | 45 |
| Target Product | 62 |
| Target Product Yield | 47 |
| Target Product Selectivity | 33 |
| Target Product Conversion Rate | 1 |
| Total tags | **492** |

**Table S5.** Entity tag counts and frequencies of each entity type in the entire corpus.

| Entity tag | Count | Frequency (%) |
| --- | --- | --- |
| Published Year | 32 | 2.04% |
| Catalytic Material | 335 | 21.38% |
| Oxidizer | 40 | 2.55% |
| Reaction Pressure | 38 | 2.43% |
| Reaction Temperature | 86 | 5.49% |
| Reaction Time | 40 | 2.55% |
| Liquid Composition and Condition | 181 | 11.55% |
| Target Product | 83 | 5.30% |
| Target Product Yield | 294 | 18.76% |
| Target Product Selectivity | 218 | 13.91% |
| Target Product Conversion Rate | 220 | 14.04% |
| Total tags | **1567** | **100%** |

**Table S6.** Shows entries extracted from 10 of the 210 publications to evaluate the LLM model’s entity extraction. Of the 110 entities, 96 had valid numerical values, all of which were accurate, and 14 were empty (“none”). This indicates 87.2% availability and nearly 100% accuracy.

| Catalyst Material | Oxidant | Reaction Pressure | Target Product | Publication Year | Reaction Temperature | Reaction Time | Conditions | Target Product Yield | Target Product Selectivity | Target Product Conversion |
| --- | --- | --- | --- | --- | --- | --- | --- | --- | --- | --- |
| Au-TiO_2_^[6]^ | O_2_ | 20 bar | CH_3_OH | 2022 | 298 K | 2 h | none | 2070.0 μmol/g | 76.80% | none |
| Au/TiO_2_^[7]^ | O_2_ | 2 bar CH_4_ 0.1 bar O_2_ | CH_3_OH | 2020 | 298 K | 2 h | water | 50 μmol | <45% | none |
| Li-MoO_x_/SiO_2_^[8]^ | O_2_ | 1 atm | HCHO | 2021 | 923 K | 1 h | deionized water null | 7611 μmol g^−1^ h^−1^ | 65% | 4.80% |
| 1% Pd/HZSM-5 (30) ^[9]^ | H_2_O_2_ | 30.5 bar | CH_3_OH | 2019 | 323.15 K | 0.5 h | H_2_O 10 g CH_4_ 30.5 bar [H_2_O_2_] 0.1 M 1500 rpm | none | 33.6 | 24 |
| Eggshell Ni/MgO-Al_2_O_3_^[10]^ | O_2_ | none | Synthesis gas | 2008 | 1073 K | 20 h | CH_4_:O_2_=1.9:1 | none | 97.10% | 95.00% |
| Cu2.0-MOR(10) ^[11]^ | O_2_ | 1 bar | CH_3_OH | 2021 | 573 K | 24 h | CH_4_ (48 mL min^-1^) + O_2_ (2 mL min^-1^) + N_2_ (50 mL min^-1^) + H_2_O(g) (0.5 g h^-1^) | 2.3 μmol | 0.34 | 0.0001 |
| PdCl_2_^[12]^ | K_2_S_2_O_8_ | 20 bar | Methyl trifluoroacetate | 2022 | 353 K | 15 h | HTFA TFAA | 7.30% | none | none |
| H_4_PV_1_Mo_11_O_40_^[13]^ | hydrogen peroxide | 50 bar | Methylformate | 2000 | 353 K | 24 h | trifluoroacetic acid anhydride | none | 72% | 4.70% |
| Cu-NU-1000^[14]^ | O_2_ | none | Methanol | 2017 | 423 K | 3 h | 50% H_2_O and 50% He | 6.9 μmol_carbon_/gCu‑NU‑1000 | 14% | none |
| DD plasma reactor^[15]^ | O_2_ | 1 bar | Methanol | 2011 | 298 K | none | CH_4_ O_2_ 50 mol% O_2_ null | none | none | 66.40% |

**References**

[1] J. Zhu, V. L. Sushkevich, A. J. Knorpp, et al. “Cu-Erionite Zeolite Achieves High Yield in Direct Oxidation of Methane to Methanol by Isothermal Chemical Looping of Article,” *Chem. Mat.* 32, no. 4 (2020): 1448-1453. 10.1021/acs.chemmater.9b04223.

[2] L. Fu, R. X. Zhang, J. L. Yang, J. L. Shi, H. Y. Jiang, J. W. Tang. “Highly Selective Conversion of CH4 to High Value-Added C1 Oxygenates over Pd Loaded ZnTi-LDH of Article,” *Adv. Energy Mater.* 13, no. 29 (2023): 10. 10.1002/aenm.202301118.

[3] A. Matsuda, K. Obara, A. Ishikawa, et al. “Bismuth phosphate nanoparticle catalyst for direct oxidation of methane into formaldehyde of Article,” *Catal. Sci. Technol.* 13, no. 18 (2023): 5180-5189. 10.1039/d3cy00590a.

[4] P. Wallis, E. Schönborn, V. N. Kalevaru, A. Martin, S. Wohlrab. “Enhanced formaldehyde selectivity in catalytic methane oxidation by vanadia on Ti-doped SBA-15 of Article,” *RSC Adv.* 5, no. 85 (2015): 69509-69513. 10.1039/c5ra10624a.

[5] K. D. Dosumov, N. M. Popova, T. S. Baizhumanova, S. A. Tungatarova. “Selective oxidation of methane into synthesis gas at short contact times on low-loading platinum-ruthenium catalysts of Article,” *Pet. Chem.* 50, no. 6 (2010): 455-461. 10.1134/s0965544110060083.

[6] L. Luo, Z. Y. Gong, Y. X. Xu, et al. “Binary Au-Cu Reaction Sites Decorated ZnO for Selective Methane Oxidation to C1 Oxygenates with Nearly 100% Selectivity at Room Temperature of Article,” *J. Am. Chem. Soc.* 144, no. 2 (2022): 740-750. 10.1021/jacs.1c09141.

[7] H. Song, X. G. Meng, S. Y. Wang, et al. “Selective Photo-oxidation of Methane to Methanol with Oxygen over Dual-Cocatalyst-Modified Titanium Dioxide of Article,” *ACS Catal.* 10, no. 23 (2020): 14318-14326. 10.1021/acscatal.0c04329.

[8] Y. Kim, T. Y. Kim, C. K. Song, et al. “Redox-driven restructuring of lithium molybdenum oxide nanoclusters boosts the selective oxidation of methane of Article,” *Nano Energy* 82, no. (2021): 9. 10.1016/j.nanoen.2020.105704.

[9] R. J. Lewis, A. Bara-Estaun, N. Agarwal, S. J. Freakley, D. J. Morgan, G. J. Hutchings. “The Direct Synthesis of H2O2 and Selective Oxidation of Methane to Methanol Using HZSM-5 Supported AuPd Catalysts of Article,” *Catal. Lett.* 149, no. 11 (2019): 3066-3075. 10.1007/s10562-019-02876-7.

[10] Y. J. Qiu, J. X. Chen, J. Y. Zhang. “A simple preparation method of eggshell Ni/MgO-Al2O3 catalyst for partial oxidation of methane of Article,” *React. Kinet. Catal. Lett.* 94, no. 1 (2008): 149-155. 10.1007/s11144-008-5236-3.

[11] A. Hirayama, Y. Tsuchimura, H. Yoshida, et al. “Catalytic oxidation of methane to methanol over Cu-CHA with molecular oxygen of Article,” *Catal. Sci. Technol.* 11, no. 18 (2021): 6217-6224. 10.1039/d1cy00676b.

[12] S. H. Cheong, D. Kim, H. T. Dang, et al. “Methane oxidation to methyl trifluoroacetate by simple anionic palladium catalyst: Comprehensive understanding of K2S2O8-based methane oxidation in CF3CO2H of Article,” *J. Catal.* 413, no. (2022): 803-811. 10.1016/j.jcat.2022.07.031.

[13] Y. Seki, J. S. Min, M. Misono, N. Mizuno. “Reaction mechanism of oxidation of methane with hydrogen peroxide catalyzed by 11-molybdo-1-vanadophosphoric acid catalyst precursor of Article,” *J. Phys. Chem. B* 104, no. 25 (2000): 5940-5944.

[14] T. Ikuno, J. Zheng, A. Vjunov, et al. “Methane Oxidation to Methanol Catalyzed by Cu-Oxo Clusters Stabilized in NU-1000 Metal-Organic Framework of Article,” *J. Am. Chem. Soc.* 139, no. 30 (2017): 10294-10301. 10.1021/jacs.7b02936.

[15] J. C. Zhou, Y. Xu, X. Zhou, et al. “Direct Oxidation of Methane to Hydrogen Peroxide and Organic Oxygenates in a Double Dielectric Plasma Reactor of Article,” *ChemSusChem* 4, no. 8 (2011): 1095-1098. 10.1002/cssc.201100093.
